# Reactive oxygen species-related oxidative changes are associated with splenic lymphocyte depletion in Ebola virus infection

**DOI:** 10.1038/s44303-025-00079-x

**Published:** 2025-04-24

**Authors:** Venkatesh Mani, Winston T. Chu, Hee-Jeong Yang, C. Paul Morris, Joseph Laux, Russell Byrum, Kurt Cooper, David X. Liu, Hui Wang, Cristal Johnson, Kyra Hadley, John G. Bernbaum, Randy Hart, Scott M. Anthony, Anthony E. Marketon, Rebecca Bernbaum-Cutler, Bapi Pahar, Gabriella Worwa, Jens H. Kuhn, Ian Crozier, Claudia Calcagno, Eric Gale

**Affiliations:** 1 Integrated Research Facility at Fort Detrick, Division of Clinical Research, National Institute of Allergy and Infectious Diseases, National Institutes of Health, Fort Detrick, Frederick, MD USA; 2 Clinical Monitoring Research Program Directorate, Frederick National Laboratory for Cancer Research, Frederick, MD USA; 3 Athinoula A. Martinos Center for Biomedical Imaging, The Institute for Innovation in Imaging, Department of Radiology, Massachusetts General Hospital, Boston, MA USA; 4 Harvard Medical School, Boston, MA USA

**Keywords:** Biomedical engineering, Magnetic resonance imaging, Molecular imaging

## Abstract

The dysregulated production of reactive oxygen species (ROS) during viral infections may lead to immune cell death and ineffective host responses. ROS dynamics have been under-investigated in severe Ebola virus disease (EVD), a condition in which hyperinflammation and excessive immune cell death are well described but poorly understood. Through ex vivo immunohistochemistry and in vivo ROS-sensitive magnetic resonance imaging (MRI) we demonstrate significant ROS-related oxidative changes in the spleens of domestic ferrets exposed to Ebola virus (EBOV). By immunohistochemistry or MRI, detection of splenic ROS was inversely correlated with the number of CD4^+^/CD8^+^ T lymphocytes and apoptotic CD8^+^ lymphocytes, but detection was positively correlated with the frequency of apoptotic CD4^+^ cells and the number and frequency of apoptotic B lymphocytes. These results suggest that ROS-induced apoptosis may contribute to the loss of splenic CD4^+^ T lymphocytes in EBOV-exposed ferrets and warrant further investigation of the role of ROS in severe EVD.

## Introduction

Ebola virus (EBOV) is a negative-sense RNA virus belonging to the *Filoviridae* family^1^ and the causative agent of Ebola virus disease (EVD) in humans^2^. EVD is an often-fatal illness^3^, with case fatality rates of 40–50% in the absence of therapeutic interventions or supportive care^4^. It initially presents with nonspecific febrile symptoms, followed by severe gastrointestinal manifestations, hematological abnormalities, and a rapid progression to multi-system organ dysfunction and damage (including the liver and kidneys)^3^. Clinical and experimental data, including from nonhuman primate (NHP) models, suggest that dysregulated immune responses, characterized by immune cell death (observed in the peripheral blood as well as bone marrow, spleen, and lymph nodes), macrophage activation syndrome (with hyperactivation of myeloid immune cells), and ineffective emergency hematopoiesis are associated with severe and fatal EVD^5–7^. Although well-described, the pathobiology of immune dysregulation during EVD is poorly understood^5–13^.

Reactive oxygen species (ROS) are important signaling mediators of cellular homeostasis and promote effective cellular immune responses to viral pathogens and other infectious agents^14–24^. However, the exaggerated release of ROS during hyperinflammatory immune responses also causes oxidative stress and has been associated with immune cell death^14,18,21–24^, which may lead to ineffective immune responses, local tissue damage, and ultimately more severe disease.

To the best of our knowledge, with the exception of limited studies reporting higher blood nitric oxide levels (another mediator related to oxidative stress) in patients with EVD^25^ or in EBOV-exposed ferrets^26^, the extent and dynamics of ROS production (and their association with immune cell loss) in humans with EVD or in experimental models of EBOV infection has not been investigated.

Here, we investigate ROS-related oxidative changes over the course of EBOV infection in domestic ferrets (a lethal model of EVD) via quantitative ex vivo immunohistochemistry (IHC) and in vivo magnetic resonance imaging (MRI) using an oxidatively activated probe called Fe-PyC3A^27–30^. Fe-PyC3A is a low-molecular-weight complex that, upon chemical oxidation, switches from a complex of divalent iron (Fe^2+^), a poor T1-relaxation agent, to a complex of trivalent iron (Fe^3+^), which is instead a strong T1-relaxation agent^27–30^, leading to MR signal enhancement in T1-weighted sequences. In a prior study, oxidative activation of Fe-PyC3A and concomitant MRI signal increase was shown to be directly linked to oxidation downstream of ROS release from myeloid cells^30^. The ex vivo and in vivo characterization of parameters related to oxidative stress in relevant organs and tissues were analyzed in relation to each other and to immune cell dynamics (in peripheral blood and spleen) and other disease markers measured in the ferret model.

## Results

### Study design

**Fig. 1:**
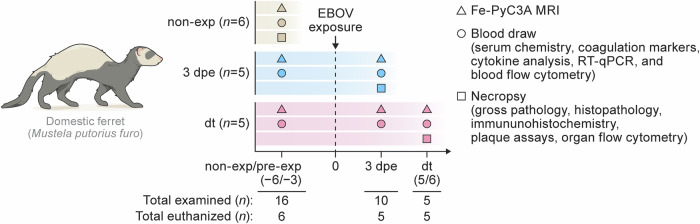
Study design. The study comprised *n* = 16 domestic ferrets, divided into three experimental groups: (a) non-exposed (non-exp, beige), ferrets that underwent Fe-PyC3A magnetic resonance imaging (MRI) and were euthanized for ex vivo readouts without viral exposure; (b) 3 dpe (blue), ferrets that underwent Fe-PyC3A MRI before Ebola virus (EBOV) exposure and at 3 dpe and were then euthanized for ex vivo readouts; (c) dt (pink), ferrets that underwent Fe-PyC3A MRI before EBOV exposure (pre-exp), at 3 dpe and at dt and were then euthanized for ex vivo readouts. 3 dpe, 3 d post-exposure; dt, day terminal, pre-exp, pre-exposure; RT-qPCR, real-time reverse transcription polymerase chain reaction.

Sixteen domestic ferrets (*Mustela putorius furo* Linnaeus, 1758; age 3–4 mo) (Supplementary Table 1) were assigned to three groups (Fig. 1): a) non-exposed controls (non-exp; *n* = 6), which were imaged and euthanized without EBOV exposure; b) 3 d post-exposure (3 dpe; *n* = 5), imaged prior to (pre-exp) and at 3 d after EBOV (variant Makona) exposure and then euthanized as per study protocol; c) day terminal (dt; *n* = 5), which were longitudinally imaged pre-exp, at 3 dpe, and once euthanasia criteria were met (dt). (See Supplementary Table 2 for the clinical scoring matrix used in the study.) Peripheral blood was sampled at pre-exp, 3 d, and dt to characterize serum chemistries, coagulation parameters, presence of EBOV RNA in plasma, and immunologic analyses over the course of the disease. Imaging was performed at the same time points. Gross pathology, histopathological and IHC analyses, as well as organ virologic and immunologic assays were performed only on the day of euthanasia (Fig. 1). (Supplementary Results, Supplementary Tables 3–7, and Supplementary Figs. 1–6).

### Clinical, virologic, and pathological analyses support a typical time course of Ebola virus disease in the domestic ferret model

**Fig. 2:**
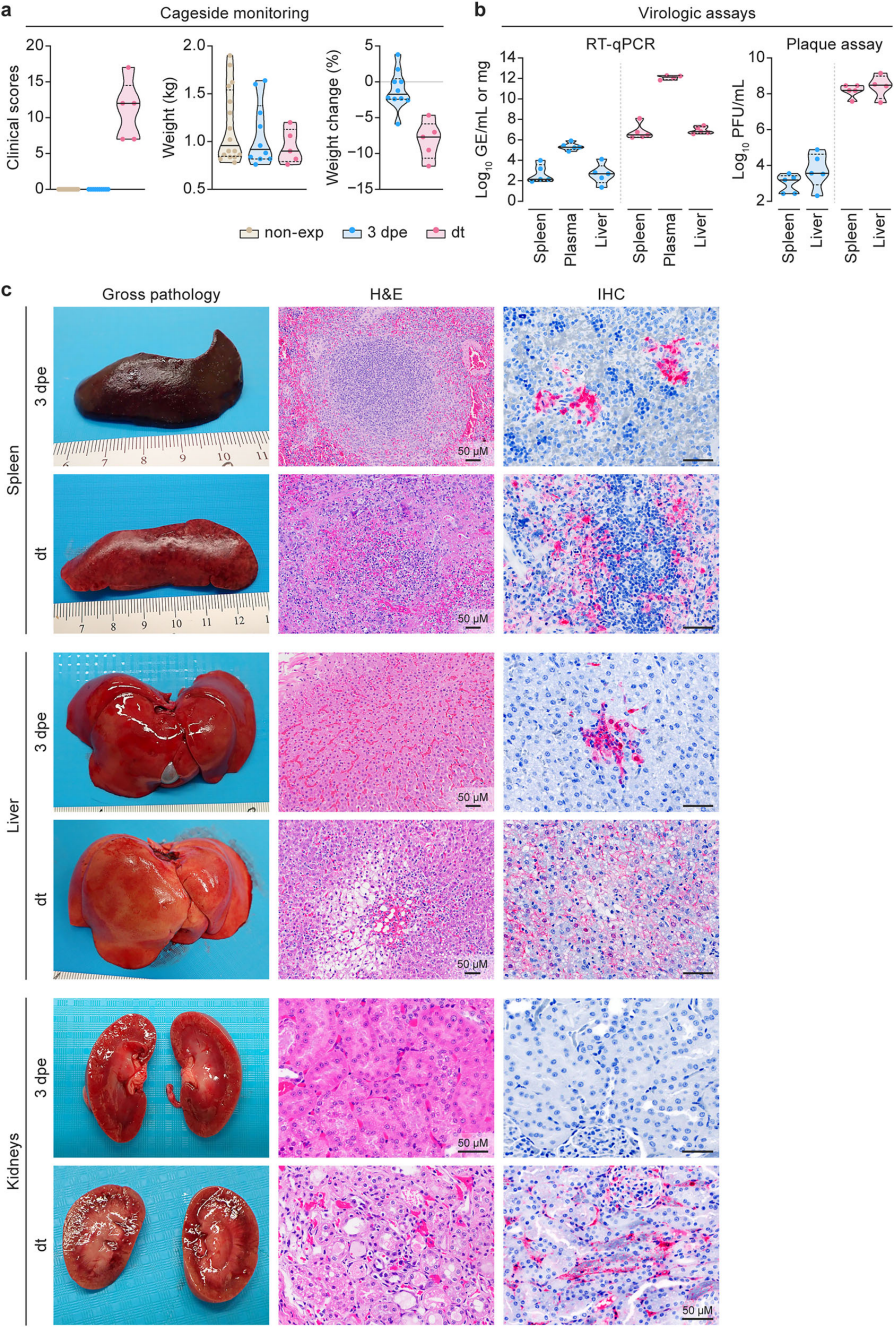
Clinical, virologic, and pathological analyses support a typical time course of Ebola virus infection in the domestic ferret model. **a** Cageside monitoring of ferrets: clinical scores (left), weight (middle), and % weight change (right) over the course of the study (Supplementary Table 3). **b** Virologic assays: real-time reverse transcription polymerase chain reaction (RT-qPCR; left) analysis of spleen, plasma, and liver tissues. Plaque assay data (right) from analysis of spleen and liver tissues (Supplementary Table 4). **c** From left to right, representative image for gross pathology, hematoxylin and eosin (H&E) histopathology, Ebola virus (EBOV) matrix protein (VP40) immunohistochemistry (IHC) in ferret spleens (top), livers (middle), and kidneys (bottom) at 3 d post-exposure (dpe) scheduled necropsy (top) and day terminal (dt, bottom).

Out of the five ferrets that were monitored until euthanasia criteria were met, one was euthanized at 5 dpe and the other four were euthanized at 6 dpe. Overall, clinical findings and results from virologic assays were in line with what has been described in EBOV-exposed ferrets^26,31–36^. Clinical cageside scores evaluated over the course of the experiments were 0 at pre-exposure baseline and at 3 dpe for all ferrets and then sharply increased to a median of 12 (interquartile range [IQR] = 7.500) at dt in EBOV-exposed ferrets, with a concomitant decrease in body weight (Fig. 2a and Supplementary Table 3). EBOV RNA, quantified using quantitative real-time reverse transcription polymerase chain reaction (RT-qPCR), was undetectable in the blood plasma, liver, and spleen samples of all ferrets before exposure but increased steadily at 3 dpe and was highest at dt, with presence of replicative virus in the livers and spleens at both post-exposure time points (Fig. 2b and Supplementary Table 4).

Major pathological findings in EBOV-exposed ferrets at dt were consistent with previous reports^26,31,35^. All the ferrets presented with macroscopical splenomegaly and histopathological expansion of red pulp with variable congestion, hemorrhage, and histiocytosis as well as lymphoid depletion and lymphocytolysis in the white pulp. By IHC, many macrophages in the red pulp and marginal zone of the white pulp tested positive for EBOV VP40 antigen (Fig. 2c, top). All ferrets also presented with hepatomegaly with friable texture at necropsy. Histopathologically, the livers were affected by multifocal coagulative necrosis admixed with hemorrhage and a small number of inflammatory infiltrates. Many Kupffer cells/macrophages and hepatocytes tested positive for EBOV VP40 antigen. (Fig. 2c, middle). Kidneys were affected by multifocal, minimal to mild interstitial nephritis, renal tubular epithelial degeneration and necrosis, and small amounts of tubular protein casts, although minimal to mild congestion was observed grossly in one ferret. Many renal interstitial macrophages and tubular epithelial cells tested positive for EBOV VP40 antigen (Fig. 2c, bottom). Other findings included multifocal variable thymic atrophy, interstitial pneumonia, and adrenal cortical degeneration and necrosis, with many macrophages in these organs testing positive for EBOV VP40 antigen. The ferrets also had macroscopical lymphadenopathy, histopathological lymphoid depletion, and lymphocytolysis, as well as many EBOV-VP40-positive macrophages in the mesenteric, axillary, and inguinal lymph nodes. Although bone marrows were grossly unremarkable, hypocellularity was revealed microscopically (Supplementary Fig. 7) with mild hemophagocytosis, megakaryocyte emperipolesis, and increased myeloid-to-erythroid (M:E) ratio. Abundant macrophages were observed, while megakaryocytes were rare, with both cell types testing positive for EBOV VP40 antigen. In contrast to organs of ferrets euthanized at dt, all organs and tissues from both unexposed controls and ferrets euthanized at 3 dpe were grossly and histopathologically unremarkable and tested negative for EBOV VP40 antigen, except that a small number of EBOV-VP40-antigen-positive macrophages were detected in one out of five axillary lymph nodes, two out of five inguinal lymph nodes, three out of five spleens, and five of five livers in the 3 dpe group.

### Ex vivo immunohistochemistry identifies an increasingly oxidative environment in the tissues of Ebola virus-exposed domestic ferrets

As a first step, we measured ROS in the ferret model of EVD using ex vivo quantitative IHC of key tissues, including the spleen, liver, and kidneys. Specific IHC markers were: (i) 4-hydroxy-2-nonenal (4-HNE), a by-product of lipid peroxidation that occurs in the presence of oxidative stress and ROS and (ii) myeloperoxidase (MPO), an enzyme typically associated with myeloid cells, and whose release in the extra-cellular space is associated with ROS production. IHC detection of 4-HNE and MPO was quantified as the percent (%) of foreground positive pixels in a defined region of interest (ROI).

**Fig. 3:**
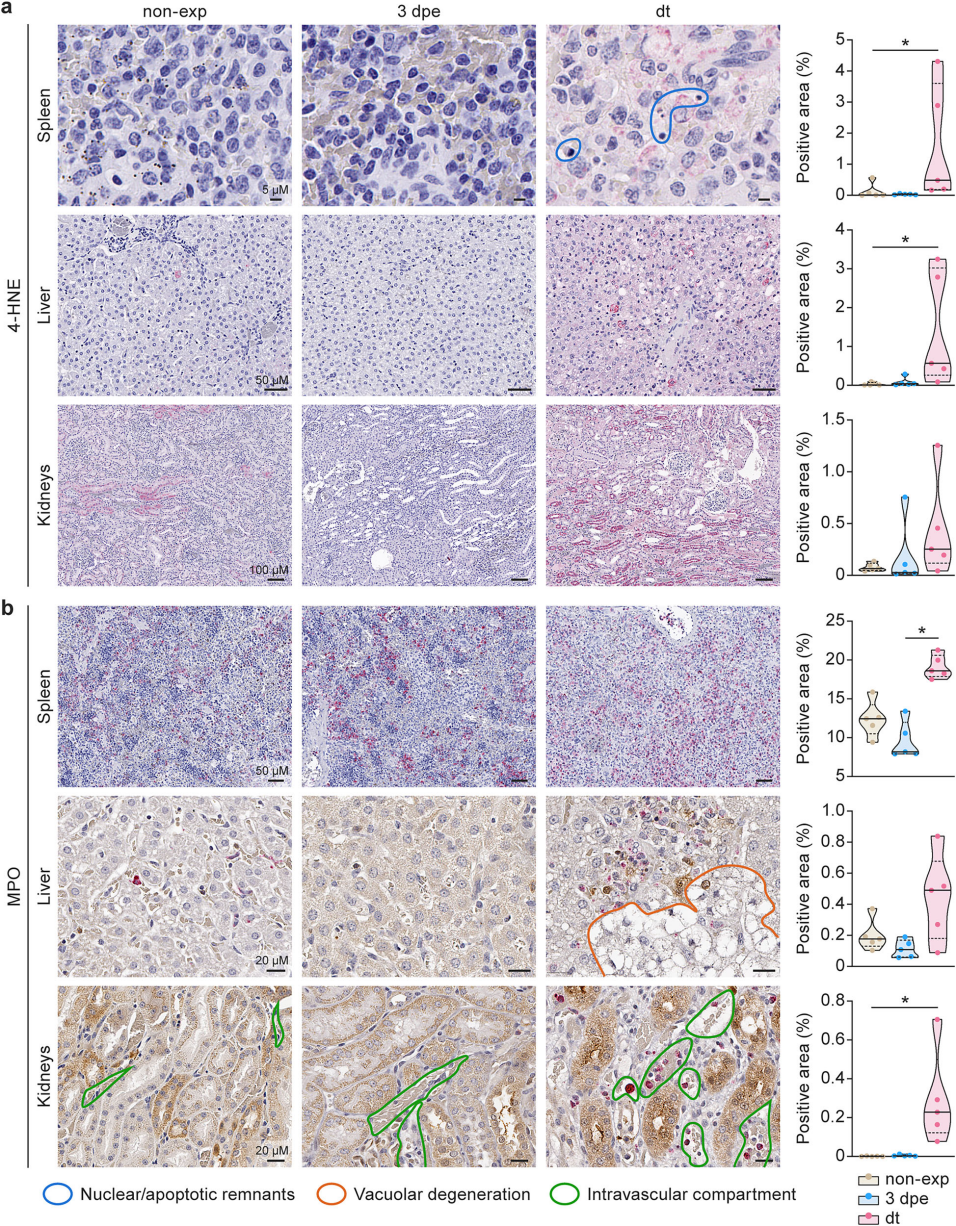
Ex vivo immunohistochemistry reveals an increasingly oxidative environment in the tissues of Ebola-virus-exposed domestic ferrets. **a** Quantitative immunohistochemistry (IHC) after 4-hydroxy-2-nonenal (4-HNE) staining of the spleen (top), liver (middle), and kidneys (bottom). **b** Quantitative IHC after myeloperoxidase (MPO) staining of spleen (top), liver (middle), and kidneys (bottom). Representative images are shown on the left, while graphs for quantitative results are shown on the right. MPO staining is indicated by red color in spleen tissue and brown color in liver and kidney tissues. Blue contours indicate nuclear/apoptotic remnants in the spleen at dt. Orange contours indicate vacuolar degeneration in the liver at dt. Green contours indicate intravascular granulocytes in the kidneys. In the graphs at the right, data from animals euthanized without viral exposure are shown at the left in beige, data from animals euthanized at 3 dpe are shown in the center in blue, and data from animals euthanized at dt are shown at the right in pink. Circles indicate individual data points. Median and interquartile range (IQR) are also represented. * indicates statistical significance (*p* < 0.05). non-exp non-exposed; 3 dpe 3 d post-exposure; dt day terminal.

In ferret spleens, the percentage of 4-HNE-positive pixels was significantly higher at dt compared to non-exposed controls (Fig. 3a, top panel), with positive pixels mainly observed in areas of cellular degeneration. A significantly higher presence of both extracellular and intracellular MPO was observed (with the percentage of positive pixels being higher at dt compared to 3 dpe, Fig. 3b, top panel), indicating an increasingly oxidizing microenvironment in the spleen over the course of the disease. In the liver, 4-HNE staining detected more lipid peroxidation in dt ferrets, particularly at borders of areas of vacuolar degeneration (Fig. 3a, middle panel). Liver MPO staining did not differ significantly among groups (Fig. 3b, middle panel, brown staining). In the kidneys, no differences across groups were found for 4-HNE (Fig. 3a, bottom panel), but there was a significant increase in the percentage of MPO-positive pixels between non-exposed controls and levels at dt (Fig. 3b, bottom panel). In the kidneys, MPO was mainly present in granulocytes in the intravascular and interstitial compartments at dt. Descriptive statistics and *p*-values for each comparison can be found in Supplementary Table 8.

### In vivo magnetic resonance imaging with Fe-PyC3A corroborates reactive oxygen species-related oxidative tissue changes identified by immunohistochemistry

To determine whether ROS-related oxidative changes could be detected longitudinally in the organs of EBOV-infected ferrets, we used MRI with the oxidatively activated probe Fe-PyC3A. In our study, Fe-PyC3A MRI acquisition and analysis focused on the spleen, liver, and kidneys (for comparison with IHC), as well as the bone marrow. Fe-PyC3A MRI was quantified by calculating the percent contrast enhancement (%CE) from before to after contrast agent injection for each imaging session.

**Fig. 4:**
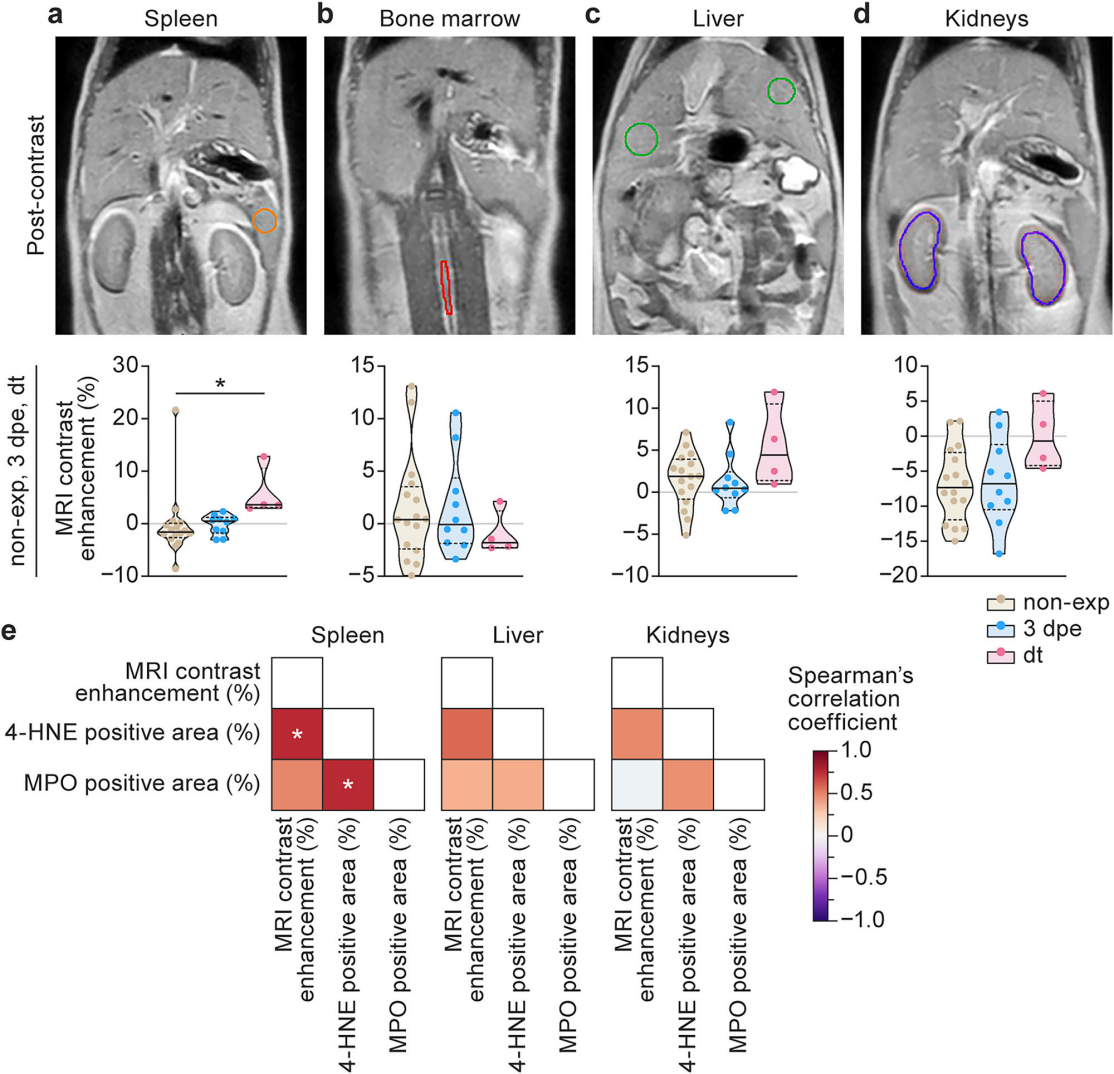
In vivo magnetic resonance imaging with Fe-PyC3A corroborates reactive oxygen species-related oxidative tissue changes identified by immunohistochemistry. **a**–**d** Representative post-contrast magnetic resonance images at dt for spleens (**a**), bone marrows (**b**), livers (**c**), and kidneys (**d**), and graphs showing the time points of the magnetic resonance imaging (MRI) readout over the course of the experiment. **e** Correlation matrices between imaging and immunohistochemistry (IHC) readouts. * indicates statistically significant comparisons (*p* < 0.05). 4-HNE 4-hydroxy-2-nonenal; MPO myeloperoxidase; non-exp non-exposed; 3 dpe 3 d post-exposure; dt day terminal.

After Fe-PyC3A injection, spleen MRI %CE ranged from mostly negative values at pre-exp to a significant increase to positive values at dt (Fig. 4a and Supplementary Fig. 6a). This pattern (also observed in other organs and tissues) can be attributed to signal loss due to a predominant T2* effect because of the presence of non-oxidized Fe-PyC3A (with very low r1 relaxivity) observed in well-perfused healthy tissues at pre-exp, as opposed to the strong predominant T1 signal enhancement observed at dt, caused by ROS-induced oxidation of Fe-PyC3A to the Fe^3+^ state (with much higher r1 relaxivity) in diseased tissues^27^.

In contrast, ROS-related %CE tended to decrease (but was not statistically significant) from pre-exp to dt in the bone marrows (Fig. 4b and Supplementary Fig. 6a). Fe-PyC3A MRI %CE in ferret livers and kidneys increased overall, although not significantly, over the course of the experiment (Fig. 4c–d and Supplementary Fig. 6a). Descriptive statistics and *p*-values for each comparison can be found in Supplementary Table 9.

The detection of ROS-related oxidative changes by Fe-PyC3A MRI was compared to ex vivo quantitative detection of 4-HNE and MPO IHC. We found positive (moderate to strong) correlations between %CE of Fe-PyC3A MRI and % positive area of 4-HNE and MPO in the spleens (though only the 4-HNE %CE was statistically significant; Fig. 4e and Supplementary Fig. 6b). Correlations between MRI %CE and 4-HNE and MPO in the livers and kidneys were weak to strong but not statistically significant (Fig. 4e and Supplementary Fig. 6c–d).

### CD4^+^ and CD8^+^ T lymphocyte death is observed in the spleens of Ebola-virus-exposed ferrets

**Fig. 5:**
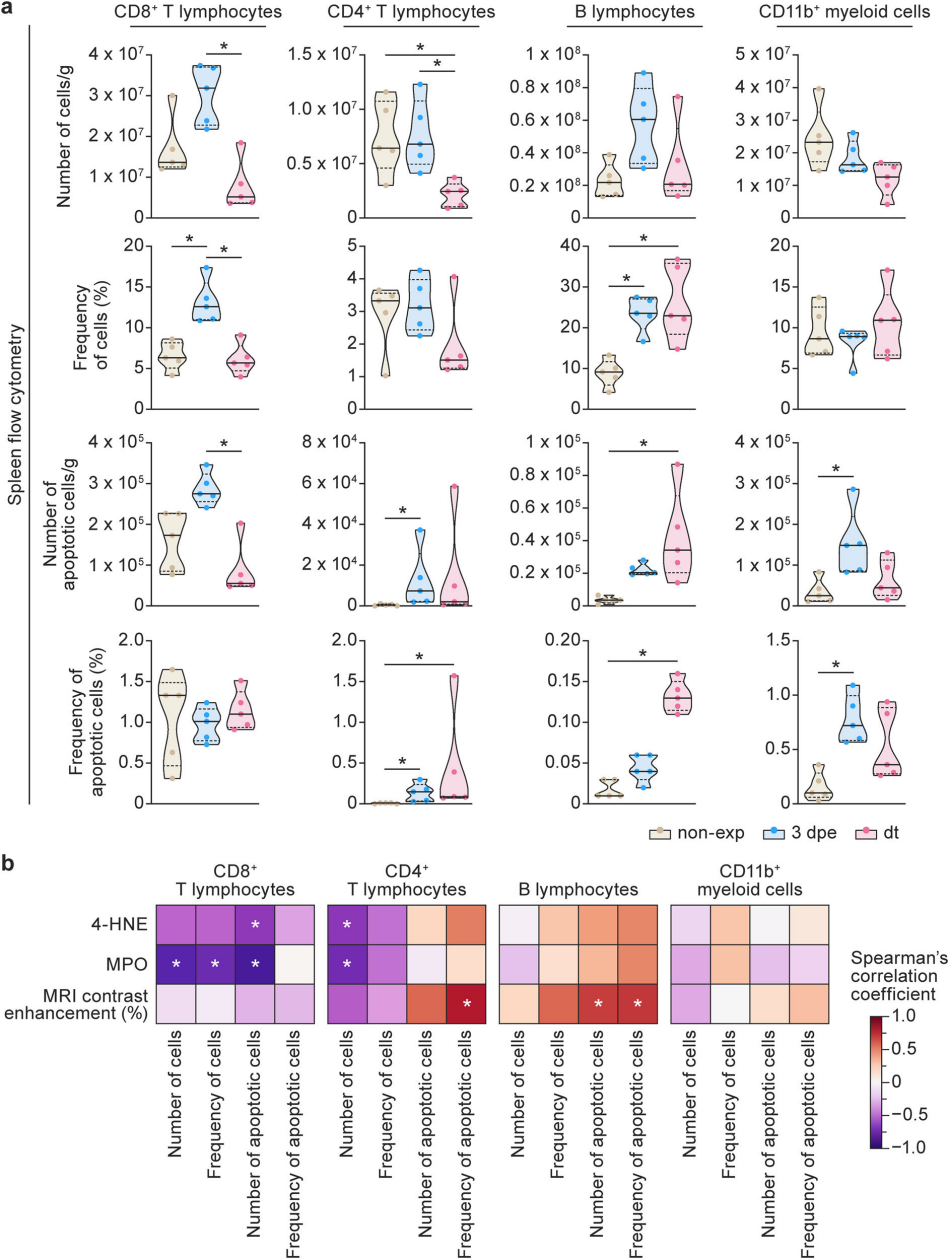
Immune cell loss in the spleens of Ebola-virus-exposed domestic ferrets is associated with an increasingly oxidative splenic environment over the course of disease after lethal-dose infection. **a** From left to right, the number and frequencies of total and apoptotic populations of CD8^+^ T lymphocytes, CD4^+^ T lymphocytes, B lymphocytes, and CD11b^+^ myeloid cells are presented over the course of the experiments. **b** Correlation matrix between imaging and immunohistochemistry and flow cytometry of the spleens (Spearman ranks). * indicates statistical significance (*p* < 0.05). 4-HNE 4-hydroxy-2-nonenal; MPO myeloperoxidase; MRI magnetic resonance imaging; non-exp non-exposed; 3 dpe 3 d post-exposure; dt day terminal.

Peripheral lymphopenia is well-described in humans with EVD^3^, and splenic lymphoid depletion has been reported in a limited number of autopsies^37^. Both have also been described in EBOV-exposed ferrets, with lymphocyte apoptosis having been observed in the spleen. Given the well-known contribution of ROS signaling to T-lymphocyte fate^38–40^, and the remarkable increase in detectable splenic ROS-related oxidative activity observed in our study (by ex vivo IHC and in vivo MRI), we used flow cytometry to characterize immune cell populations (with focus on non-granulocytes) in the spleen of the EBOV-exposed ferrets. Absolute numbers of splenic CD8^+^ and CD4^+^ T lymphocytes were relatively stable from pre-exp to 3 dpe but had significantly declined at dt (Fig. 5a), indicating local depletion of both cell populations. CD8^+^ T lymphocyte cell frequency (expressed as % of non-granulocytes) significantly increased from pre-exp to 3 dpe and then declined again to baseline levels by dt. CD4^+^ T lymphocyte cell frequency did not change over the course of the experiment. Numbers of apoptotic CD8^+^ T lymphocytes followed the same pattern found in the whole CD8^+^ T lymphocyte population, and cell frequencies (% of CD8^+^ cells) were stable over time. In contrast, CD4^+^ apoptotic cells steadily increased both in number and frequency (% of CD4^+^ cells) from pre-exp to dt (with the change in frequency being statistically significant). The absolute number of B lymphocytes was unchanged over the course of the experiment, but their relative frequency (% of non-granulocytes) increased from pre-exp to dt, despite a concomitant increase in the number and frequency of apoptotic cells. The number and frequency of splenic CD11b^+^ myeloid cells were stable over the course of disease, while apoptotic CD11b^+^ myeloid cells increased in both number and frequency from pre-exp to 3 dpe and remained stable at dt. Descriptive statistics and *p*-values for each comparison can be found in Supplementary Table 10. In conjunction with these findings, we observed significantly reduced numbers of all peripheral immune cells (CD4, CD8, B, and CD11b^+^) with increased numbers and frequency of apoptosis in peripheral CD4, CD8, and B cells, concomitantly with an increase in the concentration of several key serum cytokines at dt compared with the baseline (Supplementary Fig. 3-5 and Supplementary Tables 6-7).

### Immune cell loss in the spleens of Ebola-virus-exposed domestic ferrets is associated with an increasingly oxidative splenic environment over the course of lethal infection

Taken together, the loss of CD4^+^ and CD8^+^ T lymphocytes observed in the spleen by flow cytometry is associated with an increase in detectable ROS-related oxidative activity observed in splenic tissue by ex vivo IHC and in vivo MRI. Total numbers of CD8^+^ T lymphocytes and number of apoptotic CD8^+^ T lymphocytes were negatively correlated (with moderate to strong correlations) with percentage of 4-HNE-positive and MPO-positive pixels. Percentage of MPO-positive pixels were significantly correlated with both cell populations, while percentage of 4-HNE-positive pixels were significantly correlated only with absolute number of CD8^+^ apoptotic cells (Fig. 5b). Associations with CD4^+^ T lymphocytes showed a different pattern. We found a negative, moderate to strong correlation between spleen ROS detected by ex vivo IHC and in vivo MRI and absolute numbers of splenic T CD4^+^ lymphocytes. Correlations were stronger (with respect to MRI) and statistically significant for percent of 4-HNE-positive and MPO-positive pixels (Fig. 5b). In contrast, correlations with CD4^+^ apoptotic cells were positive (absolute numbers and fractions), stronger, and statistically significant with in vivo MRI. These flow cytometry data confirm prior histopathologic analysis indicating that splenic CD4^+^ T lymphocyte depletion is associated with apoptosis in the domestic ferret model of EVD^26^. In addition, the positive association between ex vivo IHC and in vivo MRI analysis of parameters related to oxidative stress with CD4+ lymphocyte apoptosis, suggests that ROS (either directly or via other signaling pathways) may contribute to the pathogenesis of immune dysregulation in EVD.

## Discussion

In this work, we investigated the extent and dynamics of oxidative changes related to ROS production in the lethal domestic ferret model of EVD. Oxidative changes in tissues were detected ex vivo, using IHC for 4-HNE (a by-product of lipid peroxidation) and MPO (an enzyme involved in ROS production), and in vivo, using non-invasive MRI with Fe-PyC3A (a recently developed ROS-sensitive probe)^27–30^. These approaches identified the presence of an increasingly oxidative environment in the spleens, livers, and kidneys of EBOV-exposed ferrets at terminal time points when compared to baseline. Most notably, the percent of 4-HNE-positive and MPO-positive pixels as well as % CE from in vivo MRI were all significantly increased in the spleen at dt, and ex vivo IHC (for 4-HNE) and in vivo imaging detection of ROS activity were significantly and positively correlated. The high concordance between these readouts is unsurprising, in that both techniques measure direct molecular consequences of local ROS accumulation in tissues, namely the oxidation of Fe^2+^ to Fe^3+^ of the MRI probe (which generates signal enhancement detectable by T1-weighted MRI sequences) and production of 4-HNE as a by-product of lipid peroxidation. Positive (although not statistically significant) correlations between in vivo Fe-PyC3A MRI and ex vivo IHC for MPO were also identified in spleens and livers, but not in kidneys. These apparent discrepancies may be related to differential MPO presence and activity across these organs: qualitative histopathologic examinations revealed higher levels of extracellular MPO in ferret liver and spleen (consistent with myeloid cells oxidative burst, likely leading to increase ROS production), while the majority of MPO observed in the kidneys was intracellularly located within an intravascular population of granulocytes (and thus unlikely be linked to ROS production in tissue).

Overall, the increasingly oxidative environment observed over the course of the disease (either by ex vivo IHC or in vivo imaging) was concomitant with the development of a typical peripheral inflammatory response and a dramatic loss of CD8^+^ and CD4^+^ T lymphocytes, B lymphocytes and CD11b^+^ myeloid cells in the peripheral blood, as previously well-documented in EVD patients and NHP models of EVD^41–43^. Regarding the spleen, histopathology from human (albeit from limited sampling) disease and in NHP and ferret models suggests that a significant depletion of lymphoid populations is characteristic of severe EVD. Our approach, combined with flow cytometry, confirmed these findings in the ferret model and identified a significant association between ROS-related oxidative changes and decreased overall numbers (with increased apoptosis) of splenic CD4^+^ T lymphocytes. Although the pathogenesis of splenic immune cell loss in EVD is unknown, our findings, given the known role for ROS signaling in determining T lymphocyte fate in physiological and disease states^44^, suggest a role for ROS-related apoptotic cell death in the depletion of splenic CD4^+^ T lymphocytes (but not splenic CD8^+^ T lymphocytes) in EVD. Known differences in ROS-induced metabolic changes between CD4^+^ and CD8^+^ T lymphocytes^45^ and different degrees of susceptibility to ROS-induced apoptosis in different kinds of T lymphocytes observed in other disease settings may offer an explanation for this finding^46–48^. In parallel to lymphoid depletion in the spleen (and peripheral blood), we observed a significant increase in serum TNF levels (among other cytokines) over the course of the study. TNF is a master regulator of cell survival and death, and an increase in TNF can promote ROS production through the NADPH oxidase enzyme pathway^49^. The possible crosstalk between increased TNF production, excessive ROS accumulation, and immune cell death in EVD and any impact on disease severity and progression needs further investigation.

These novel insights into the association between organ-specific ROS-related oxidative changes and lymphoid depletion in EVD were enabled by significant technical developments implemented in this study. Domestic ferrets are often used in studies of infectious agents^34,50–52^ and serve as a well-established small-animal model of filovirus disease^26,31,33,35,36,43,53–59^. In contrast to prior experiments utilizing the ferret model of EVD, we applied in vivo imaging with a recently developed molecular imaging probe (Fe-PyC3A), combined with IHC detection to explore the role of ROS-related oxidative changes at the tissue level, focusing on long-recognized (but poorly understood) immune cell death. Compared to ex vivo snapshot readouts, in vivo imaging enables non-invasive monitoring of organ pathophysiology in the same experimental animals over time. These advantages have implications for the ability to design in vivo imaging studies in infectious disease models using a lower number of animals, while mitigating concerns about statistical variability and maintaining adequate statistical power^26^. Because of the significant technical challenges in installing and using medical imaging equipment (pre-clinical and clinical) in a maximum-containment setting, the use of non-invasive imaging to evaluate disease course and pathogenesis of risk group 4 pathogens has, so far, been uncommon^60^. Previous imaging studies in animal models have targeted evaluation of liver^61,62^, brain^63^, and ocular^64^ involvement and host responses^65^ in either EBOV-exposed or Marburg-virus-exposed NHPs using MRI^63,64^, computed tomography (CT)^62^, or positron emission tomography (PET)^65^. In the clinical setting, CT and (limited) MR imaging has been used to investigate neurological sequelae in EVD survivors^66–68^, and MRI has been used to evaluate myocarditis in one acute EVD case^69^. To our knowledge, ours is the first imaging study in a small-animal model of EVD that uses in vivo molecular imaging to probe aspects of host responses thought to be related to severe EVD and to relate non-invasive imaging findings to specific immunological dysregulations in this context.

In addition, although IHC detection of ROS via 4-HNE and MPO IHC is established, application to any animal model of EVD is novel, and the quantification of these parameters using percentage of positive pixels was newly developed for this study. Finally, although serum cytokine measurements and flow cytometric analyses have been used to characterize EVD in patients and in NHP models^41–43^, these measurements are newly applied to the ferret model (one study has evaluated cytokine RNA transcripts in EBOV-exposed ferrets^43^).

While our results suggest an intriguing association between tissue ROS-related oxidative changes and lymphocyte depletion typical of severe EVD (specifically in the spleen), limitations of our study preclude a more comprehensive understanding of the role of ROS (and oxidative stress in general) in the pathogenesis of this complex disease. The number of ferrets included in the study was small at origin (*n* = 16 total, limited by the logistics of operating in a biosafety level 4 [BSL-4] environment) and was further reduced over the course of the study (with only *n* = 5 ferrets reaching dt), thereby restricting ex vivo analyses at different time points after exposure. Additionally, in all ferrets, ex vivo analyses were performed after injection of Fe-PyC3A (with some of the unexposed animals injected multiple times with escalating doses to establish the optimal dose for in vivo imaging). Other technical limitations precluded a more comprehensive interpretation of results deriving from IHC and immunologic assays. For example, in our experiments, cellular morphology assessed from IHC suggests that the majority of intracellular MPO observed at dt is present within macrophages. However, the identity of cells containing intracellular MPO (and the source of extracellular MPO) could not be further and unequivocally confirmed using flow cytometry because of a lack of commercially available surface markers specific for other relevant MPO-producing cell types, such as granulocytes/neutrophils, in the ferret model. Another acknowledged major limitation is the lack of 4-HNE and MPO IHC, as well as flow cytometry analysis of the bone marrow (and lymph nodes). The absence of these quantitative readouts makes it challenging to interpret the time course of the bone marrow Fe-PyC3A MRI signal over the course of the study. However, a significant decrease in MPO^+^ cells reported in the bone marrow of EBOV-exposed NHPs starting from 3 dpe^6^, and our histopathological analyses, confirming hypocellularity of the ferret bone marrow at dt (see Supplementary Results and Supplementary Fig. 7), suggest that the decrease in Fe-PyC3A MRI signal in this tissue may be related to a loss of MPO-producing cells (and consequent decreased ROS production) over the course of the disease. This finding may be suggestive of ineffective emergency hematopoiesis (as described in EVD patients^7^ and previous NHP studies^6^), and it may offer an explanation for the loss of immune cells observed in the peripheral blood over the course of the experiment (see Supplementary Results and Supplementary Fig. 3–5).

In conclusion, our study provides novel insight into a potential role for ROS-related oxidative changes in the pathogenesis of EVD, specifically in the depletion of key immune cell populations, that urges further investigation. Furthermore, we highlight the utility of medical imaging in the characterization of key molecular and cellular processes in infectious diseases. We foresee future experiments (in the ferret or other small-animal models of EVD) that capitalize on the combination of Fe-PyC3A MRI (or other non-invasive in vivo imaging readouts) and ex vivo histopathologic and immunologic analyses to further investigate the role of immune organs (including the bone marrow) and immune mediators (other than ROS) in determining the pathogenesis and outcomes of severe EVD.

## Methods

### Study design and clinical monitoring

The experiments were performed in the maximum BSL-4 containment laboratory at the Integrated Research Facility at Fort Detrick (IRF-Frederick). The facility is accredited by the Association for Assessment and Accreditation of Laboratory Animal Care International (AAALAC). The IRF-Frederick is part of the National Institutes of Health (NIH), the National Institute of Allergy and Infectious Diseases (NIAID), and the Division of Clinical Research (DCR). Experimental procedures were approved by the NIAID DCR Animal Care and Use Committee (ACUC) and conducted in compliance with the Animal Welfare Act regulations, Public Health Service policy, and the Guide for the Care and Use of Laboratory Animals (Eighth Edition).

The study included 16 domestic ferrets (*Mustela putorius furo* Linnaeus, 1758; age, 3–4 mo; weight, 0.78–1.9 kg at the start of the study; Supplementary Table 1). Ferrets were housed by sex, with one or two animals per cage. Prior to study initiation, ferrets were acclimatized for approximately 2 weeks and health assessments were performed by veterinary staff.

Six ferrets (non-exposed controls) were imaged and euthanized without viral (or mock) exposure to establish baseline characteristics (Fig. 1). Five ferrets (3 dpe group) were imaged before viral exposure and at 3 d post-exposure and then euthanized (Fig. 1). The remaining five ferrets (dt group) were imaged before viral exposure and at 3 d and were then monitored until they reached euthanasia criteria (Supplementary Table 2), when they were imaged again and then euthanized (Fig. 1). Exposed ferrets were inoculated with EBOV variant Makona (1.98E^+ 03^ ± 589.2 PFU of Ebola virus/H. sapiens-tc/GIN/2014/Makona-C05, 0.3 mL volume), injected subcutaneously into the mid-back. Inoculation doses were confirmed by back-titration using plaque assays^70^. Ferrets were observed and assigned a clinical score at the cageside from pre-exp through dt (Supplementary Table 2). Weights were also monitored.

### Virologic assays and assessment of hematology, serum chemistry, blood coagulation and proinflammatory markers

The presence of EBOV RNA was detected longitudinally in sera from samples attained at pre-exp, 3 d, and dt (at euthanasia), as well as in liver and spleen tissue samples, using RT-qPCR^70^. The presence of replicative virus in the livers and spleens at the time of euthanasia was confirmed by plaque assays^70^. Serum chemistries were measured with Piccolo General Chemistry 13 panels (Abaxis, Union City, CA, USA). Coagulation parameters were measured using a CA-660 analyzer (Siemens, Malvern, PA, USA).

### Gross pathology, histopathology, and immunohistochemistry for viral proteins

Complete postmortem examinations were performed on all ferrets, and gross findings were documented. Tissues were collected in bead beat tubes for virologic assays or trimmed (thickness, <1 cm), placed in cassettes, and fixed for more than 72 h in 10% neutral-buffered formalin for histopathology and IHC assays. Microscopic examination of routinely prepared hematoxylin-and-eosin-stained paraffin sections was performed by light microscopy and observations were scored by severity. IHC for viral proteins using primary mouse anti-EBOV VP40 antibody (IBT Bioservices, Rockville, MD, USA) was performed on formalin-fixed, paraffin-embedded, sectioned (thickness, 4 mm) tissues. The biotinylated target was detected by a VECTASTAIN ABC-AP kit, and positive staining was visualized with ImmPACT Vector Red AP Substrate Kit chromogen as previously described^71^.

### Isolation and staining of splenocytes and acquisition of flow cytometry data in the peripheral blood and spleen

Spleens were weighed and 400–500 mg of tissue were homogenized in c-tubes using a gentleMACS Dissociator (Miltenyi Biotec, Gaithersburg, MD, USA). After dissociation, homogenates were washed with 2% heat-inactivated fetal bovine serum (Sigma-Aldrich, St. Louis, Missouri, USA) + phosphate-buffered saline (PBS; Gibco, Grand Island, NY, USA) + 2 mM ethylenediamine tetraacetic acid (Invitrogen, Carlsbad, CA, USA) (PBS-2), strained through a 100-µM filter (Miltenyi Biotec), lysed with ammonium-chloride-potassium (Quality Biological, Gaithersburg, MD, USA), and counted on a Cellometer automated cell counter (Nexcelom Bioscience, Lawrence, MA, USA). Isolated cells were washed with PBS-2 and diluted to the appropriate concentration prior to proceeding to staining for flow cytometry. Whole blood (100 µL of unlysed whole blood) or isolated splenocytes (up to 2.5 × 10^6^ isolated cells), resuspended to a volume of 100 µL of PBS-2, were blocked with 5 µL of Fc block (Clone 2.4G2; BD Biosciences, Franklin Lakes, NJ, USA) for 10 min on ice to inhibit Fc receptor-specific binding. Blocked samples were stained in total volumes of 200 µL with a mixture of conjugated primary antibodies including CD11b BV510 (clone M1/70; Biolegend, San Diego, CA, USA), CD25 Alexa Fluor 647 (clone 7D4; BD Biosciences, Franklin Lakes, NJ, USA), HLA-DR BV650 (clone L243; Biolegend), F4/80 BV711 (clone BM8; Biolegend), CD4 FITC (clone 02; Sino Biological US Inc., Wayne, PA, USA), CD11a PE (clone 2D7; Biolegend), and CD8a PerCP (clone Lyt2; Sino Biological US Inc.), amine-reactive dye (Live Dead Blue; Thermo Fisher Scientific, Waltham, MA, USA) for dead cell exclusion, and Brilliant Stain Buffer Plus (BD Biosciences) for 20–30 min on ice. Samples were washed, fixed, and inactivated for at least 30 min with a minimum of 500 µL of Cytofix/Cytoperm (BD Biosciences), washed with 1X Perm wash (BD Biosciences), blocked intracellularly with 5 µL of Fc block for 10 min on ice, and stained in a total volume of 150 µL with a mixture of primary antibodies against intracellular targets including Cleaved Caspase 3 PE-Cy7 (clone D3E9; Cell Signaling Technology, Denvers, MA, USA), and CD79a PerCP-Cy5.5 (clone HM47; Biolegend) for 30 min on ice. Fully stained inactivated samples were then washed with 1X Perm wash, resuspended in PBS, and assessed on a five-laser Aurora Flow Cytometer (Cytek Biosciences, Fremont, CA, USA) on low or medium flow rate settings (Supplementary Fig. 2). For both whole blood and splenocytes, an identical flow cytometric gating strategy was used to delineate distinct subsets of cell populations (Supplementary Fig. 2). Data analysis was focused on non-granulocyte populations. Flow data were analyzed using FlowJo software version 10.10.

### Host chemokine/cytokine protein multiplex analysis

Host cytokines were measured on a Flexmap 3D Multiplexing System (Luminex Corporation, Austin, TX, USA). Analytes in the Ferret Cytokine Panel 1 Luminex 12-plex (F103-K) included IFNA, IL12B, IL12, IL6, CXCL8, CXCL10, CCL2, CCL4, IL2, TNF, IL4, and IL17 (Ampersand Biosciences, Lake Clear, NY, USA). Sera were isolated from whole blood samples and stored at −80 °C until testing. Briefly, sera collected at baseline and indicated time points from unexposed control animals or virus-exposed animals were added to magnetic beads conjugated to antibodies that target the specified analytes. After a series of washes, secondary antibodies conjugated to phycoerythrin were added to the magnetic beads and loaded into the Flexmap 3D instrument for analysis with the appropriate kits. Test kits were used in accordance with the manufacturer’s protocol. Raw data were exported as .csv files and analyzed in Excel using Bioplex Results Generator 3.0 and Bioplex Manager 6.1. Concentrations of each analyte were based on the standard curve generated from standards provided by the manufacturer. All values were normalized to sera values per ferret at pre-exp baseline time points.

### 4-hydroxy-2-nonenal immunohistochemistry

Formalin-fixed paraffin-embedded tissue sections were deparaffinized and rehydrated through a series of treatments with graded ethanol. The sections were put through antigen retrieval using Diva Decloaker citrate buffer (cat# DV2004; Biocare Medical, Pacheco, CA) for 15 min at 95 °C to unmask antigens. After rinses with tris-buffered saline with TWEEN (TBST), an Avidin/Biotin Blocking Kit (SP2001; Vector Laboratories, Inc. Newark, CA) was used. The Avidin block solution was applied for 15 min at room temperature (RT), followed by a rinse in TBST. A 15-min room temperature (RT) incubation with biotin block was added, followed by another TBST rinse. BLOXALL Endogenous Blocking Solution (cat# SP6000; Vector Laboratories, Inc.) was applied for 10 min at RT, followed by a TBST rinse. A protein block, Background Sniper (cat# BS966; Biocare Medical), was applied for 30 min at RT. Sections were then incubated with a 4-HNE primary antibody (cat# MHN-100P; Japan Institute for the Control of Aging [JaICA] Nikken SEIL Co., Ltd., Fukuroi, Shizuoka, Japan) at a concentration of 0.2 µg/mL for 20 h at 4 °C. The sections were rinsed with TBST, followed by incubation in a biotin-conjugated donkey anti-mouse secondary antibody (cat# 715-065-151; Jackson ImmunoResearch Laboratories, West Grove, PA) for 60 min at RT. The sections were rinsed with TBST, and the biotin label was detected by a 30-min RT incubation using the VECTASTAIN ABC-AP Kit (cat# AK-5000; Vector Laboratories, Inc.). The sections were again rinsed with TBST, and positive staining was visualized after a 20-min RT incubation using the ImmPACT Vector Red Substrate Kit, Alkaline Phosphatase (cat# SK-5105; Vector Laboratories, Inc.). The slides were then counterstained with hematoxylin, dehydrated, and coverslipped.

### Myeloperoxidase immunohistochemistry

Formalin-fixed paraffin-embedded tissue sections were deparaffinized and rehydrated through a series of treatments with graded ethanol. The sections went through a retrieval process to unmask antigens using Diva Decloaker citrate buffer (cat# DV2004; Biocare Medical) at 120 °C for 30 s and then left alone to cool to 95 °C. After rinses with TBST, endogenous alkaline phosphatase was blocked with BLOXALL Endogenous Blocking Solution (cat# SP6000; Vector Laboratories, Inc.) for 10 min at RT, followed by a TBST rinse. Background Sniper (cat# BS966, Biocare Medical), was applied for 30 min at RT. Sections were then incubated using a rabbit anti-MPO primary antibody at a dilution of 1:100 (cat # PA5-16672; Thermo Fisher Scientific, Waltham, MA, USA) for 20 h at 4 °C. The sections were rinsed with TBST, followed by a 60-min incubation at RT using a biotin-conjugated donkey anti-rabbit secondary antibody (cat# 715-065-151; Jackson ImmunoResearch Laboratories). The sections were again rinsed with TBST, and the biotin label was detected by a 30-min RT incubation of VECTASTAIN ABC-AP Kit (cat# AK-5000; Vector Laboratories, Inc.). Positive staining was visualized with a 20-min RT incubation using ImmPACT Vector Red Substrate Kit, Alkaline Phosphatase (cat# SK-5105; Vector Laboratories, Inc.). The slides were then counterstained with hematoxylin, dehydrated, and coverslipped.

### Double macrophage and myeloperoxidase staining

Formalin-fixed paraffin-embedded tissue sections were deparaffinized and rehydrated through a series of graded ethanol. The sections went through antigen retrieval to unmask the antigens using Diva Decloaker citrate buffer (cat# DV2004; Biocare Medical) at 95 °C for 30 min. After rinses with TBST, Background Sniper (cat# BS966, Biocare Medical), was applied for 60 min at RT. A biotin-labeled rat-anti-MAC-2 primary antibody at a concentration of 0.67 µg/mL (cat# CL8942B; Cedarlane, Burlington, NC) was added and incubated for 60 min at RT. The sections were rinsed with TBST and endogenous peroxidase was blocked with Peroxidazed 1 (cat# PX968; Biocare Medical) for 10 min at RT. The sections were rinsed with TBST, and the biotin label was detected by the addition of VECTASTAIN Elite ABC-HRP Kit, Peroxidase (cat# PK-6100; Vector Laboratories, Inc. Newark, CA) for 30 min at RT. The slides were rinsed with TBST, and the positive staining was visualized after a 5-min RT incubation with the Betazoid DAB Chromogen Kit (cat# BDB2004; Biocare Medical). The sections were again rinsed with TBST, and endogenous alkaline phosphatase was blocked using BLOXALL Endogenous Blocking Solution (cat# SP6000; Vector Laboratories, Inc. Newark, CA) for 10 min at RT. Slides were rinsed with TBST and Background Sniper (cat# BS966; Biocare Medical) was applied for 30 min at RT. Sections were then incubated using a rabbit anti-MPO primary antibody at a dilution of 1:150 (cat# PA5-16672; Thermo Fisher) for 60 min at RT. The sections were rinsed with TBST, followed by a 30-min incubation at RT using a biotin conjugated donkey anti-rabbit secondary antibody (cat# 715-065-151; Jackson ImmunoResearch Laboratories). The sections were rinsed with TBST and the biotin label was detected by a 30-min RT incubation of the VECTASTAIN ABC-AP Kit (cat# AK-5000; Vector Laboratories, Inc.). The sections were rinsed with TBST and positive staining was visualized with a 20-min RT incubation of the ImmPACT Vector Red Substrate Kit, Alkaline Phosphatase (cat# SK-5105; Vector Laboratories, Inc.). The slides were then counterstained with hematoxylin, dehydrated, and coverslipped.

### Analysis of immunohistochemistry images

After staining and digital acquisition of IHC slides, 4-HNE-positive and MPO-positive areas were identified and quantified by color-deconvolution using a set of custom programs written in Python programming language (using Python libraries skimage, scipy, shapely, and streamlit) and QuPath^72^, an open-source software for the analysis of histopathology images. First, tissue regions of interest (ROIs) were manually annotated on digital histopathology slides by a board-certified pathologist to exclude tissues other than from the target organ (e.g., gallbladder in the case of the liver). Next, image resolution for each digitized slide was down-sampled by a factor of 4 by local averaging and smoothed with a Gaussian blur (sigma = 1). White-glass pixels with values >220 for red, >220 for green, and >220 for blue were excluded from the tissue ROI. Multiple methods of color-deconvolution were attempted on image patches from representative slides from each slide set until satisfactory color deconvolution was achieved. To generate an accurate representation of the individual stains, for 4-HNE, stain vectors were instead calculated using QuPath’s “estimate stain vectors” tool applied to three separate, single-stain, control slides (red, 4-HNE; blue, hematoxylin; and brown, 3,3′-diaminobenzidine [DAB]). The published hematoxylin-eosin-DAB stain vectors^73^ provided the best color deconvolution for MPO staining. The chosen stain vectors were used to perform color deconvolution^73^ on the 4-HNE and MPO IHC images. Thresholds were then applied to obtain masks of 4-HNE-positive or MPO-positive pixels. The channel absorbance thresholds were determined by a board-certified pathologist. After identification of 4-HNE-positive and MPO-positive areas, results were expressed as the percent positive pixels in the ROI as follows:

$$\% {positive\; pixels}=100\cdot \quad {positive\; pixels}/{total\; pixels}$$

### Magnetic resonance image acquisition

Ferrets were imaged on a 3 T Achieva MRI scanner (Philips Healthcare, Andover, Massachusetts, USA) using an 8-channel knee coil for signal reception. Animals were anesthetized with an intramuscular injection of glycopyrrolate (0.01 mg/kg, Hikma Pharmceuticals, Berkeley Heights, NJ), ketamine (10 mg/kg, Dechra Veterinary Products, Overland Park, KS), and dexmedetomidine (0.08 mg/kg, Covetrus North America, Dublin, OH) and intubated to enable breath holds during the imaging session. Anesthesia was maintained for the imaging session with isoflurane (1–3%, Covetrus North America, Dublin, OH). A 24-gauge intravenous catheter was placed in the cephalic or saphenous vein for Fe-PyC3A contrast agent injection. Fe-PyC3A is the iron complex of *N*-picolyl-*N*,*N*′,*N*′-*trans*-1,2-cyclohexyleneiaminetriacetic acid. Fe-PyC3A r1 relaxation rates in the Fe^2+^ and Fe^3+^ oxidation states are, respectively, 0.18 mM^–1^s^–1^ at and 2.4 mM^–1^s^–1^ at room temperature and at 4.7 Tesla (T). For this study, Fe-PyC3A produced in-house (E. G. laboratory, Harvard Medical School, Boston, MA, USA), shipped to the Integrated Research Facility at Fort Detrick in powder form, and then reconstituted right before imaging. After localizer scans, two-dimensional (2D) T1-weighted single shot (SS) fast spin echo (FSE) coronal images of the ferret abdomens were acquired using respiratory triggering, with the following sequence parameters: repetition time (TR), 750 ms; echo time (TE), 20 ms; echo train length (ETL), 32; signal average, 1; slice thickness, 2 mm; slice gap, 1 mm; number of slices, 24; interpolated in-plane spatial resolution, 0.375 × 0.375 mm^2^.

After acquisition of pre-contrast images, Fe-PyC3A was injected manually through the intravenous catheter (concentration, 0.05 mmol/kg). Post-contrast 2D T1-weighted SS FSE images were acquired 30 ± 7 min after contrast agent injection, with the same imaging parameters used to acquire pre-contrast images. After a terminal imaging session, while still under anesthesia, ferrets were euthanized by intravenous/intracardiac injection of a barbiturate euthanasia solution (pentobarbital 390 mg/mL, 0.1–0.2 mg/kg) administered intravenously, to undergo post-mortem procedures.

### Magnetic resonance image analysis

After acquisition, MRI data were transferred to a workstation with MIM software (version 7.2.9) for image analysis. ROIs were traced on coronal views of the spleens, livers, kidneys, and bone marrows. One 3D ROI was traced in the spleen, using a 3-dimensional (3D) brush tool with an 8-mm diameter. Two 3D ROIs were traced in the liver (one in the left lobe and one in the right lobe^74^), using a 3D brush tool with a 12-mm diameter. For the left and right kidneys, one 2D ROI was traced for each coronal slice in which the kidneys were visible, encompassing the whole organ. One 2D ROI was traced in the bone marrow (lumbar vertebrae, just below the kidneys^75^), using a rectangular contour of 18–22 mm in length, manually adjusted to stay within the bone marrow boundaries. Spleen and liver ROIs were traced by two observers (H.W. and H.J.Y., both with more than 5 yr experience in analyzing medical images); kidney and bone marrow ROIs were traced by one observer (H.J.Y.). Kidney ROIs were averaged across left and right kidneys before statistical analysis was conducted. Liver ROIs were averaged across left and right lobes. Results from different observers were also averaged when available. To evaluate ROI enhancement after Fe-PyC3A injection, %CE between pre- and post-contrast agent injection images was evaluated using this equation:

$$\% {CE}=100\cdot \quad \left({{SI}}_{{post}}-{{SI}}_{{pre}}\right)/{{SI}}_{{pre}}$$

with SI being the MRI signal intensity in a given ROI, *pre* indicating pre-contrast images and *post* indicating post-contrast images.

### Statistical analysis

Statistical analyses were performed using Prism version 10.2.2 (GraphPad). Given the relatively low number of animals in this study, unpaired, non-parametric, two-tailed statistical tests (either Mann-Whitney for two groups, or Kruskal–Wallis for more than two groups) were used to compare measurements across different groups of ferrets. Corrections for multiple comparisons were applied using Dunn’s test. Spearman’s rank was used to explore correlations between different measurements. Correlations with a Spearman’s rank correlation coefficient (*R*) of 0 ≤ |*R* | < 0.3 were deemed to be of low strength, correlations with 0.3 ≤ |*R* | < 0.7 were deemed to be of moderate strength, and correlations with 0.7 ≤ |*R* | ≤ 1 were deemed to be strong. Correlation *p*-values were corrected for the false discovery rate using the Benjamini–Hochberg procedure^76^.

## Supplementary information

Supplementary figures

## Data Availability

Data that support the findings of this study are available from the corresponding author on reasonable request.

## Code availability

Programming code used for data analysis is available from the corresponding authors on reasonable request.
