# Author Correction: Reactive oxygen species-related oxidative changes are associated with splenic lymphocyte depletion in Ebola virus infection

**DOI:** 10.1038/s44303-025-00091-1

**Published:** 2025-06-02

**Authors:** Venkatesh Mani, Winston T. Chu, Hee-Jeong Yang, C. Paul Morris, Joseph Laux, Russell Byrum, Kurt Cooper, David X. Liu, Hui Wang, Cristal Johnson, Kyra Hadley, John G. Bernbaum, Randy Hart, Scott M. Anthony, Anthony E. Marketon, Rebecca Bernbaum-Cutler, Bapi Pahar, Gabriella Worwa, Jens H. Kuhn, Ian Crozier, Claudia Calcagno, Eric Gale

**Affiliations:** 1 Integrated Research Facility at Fort Detrick, Division of Clinical Research, National Institute of Allergy and Infectious Diseases, National Institutes of Health, Fort Detrick, Frederick, MD USA; 2 Clinical Monitoring Research Program Directorate, Frederick National Laboratory for Cancer Research, Frederick, MD USA; 3 Athinoula A. Martinos Center for Biomedical Imaging, The Institute for Innovation in Imaging, Department of Radiology, Massachusetts General Hospital, Boston, MA USA; 4 Harvard Medical School, Boston, MA USA

**Keywords:** Biomedical engineering, Magnetic resonance imaging, Molecular imaging

Correction to: *npj Imaging* 10.1038/s44303-025-00079-x, published online 24 April 2025

In this article the ‘Conflict of Interest’ statement was incorrectly given as ‘The authors declare no competing interests’ but should have been ‘E.M.G holds equity and receives consulting income from Reveal Pharmaceuticals, a company that is developing manganese-based MRI contrast agents’. The original article has been corrected”.

