# Supplementary material for: Reactive oxygen species-related oxidative changes are associated with splenic lymphocyte depletion in Ebola virus infection
Source: Npj Imaging. 2025 Apr 24;3:16. doi: 10.1038/s44303-025-00079-x (PMC12021656; doi:10.1038/s44303-025-00079-x)

Supplementary Results

Serum chemistry and coagulation markers support a typical time course of Ebola virus infection in the domestic ferret model

As expected, concentration of liver enzymes (alanine transferase [ALT], aspartate aminotransferase [AST] and gamma-glutamyl transferase [GGT]), as well as total bilirubin, alkaline phosphatase (ALP) and amylase, significantly increased over the course of the study. Among markers of kidney function, blood urea nitrogen (BUN) and creatinine (CREA) concentrations were also significantly increased. Coagulation assays confirmed the abnormalities usually encountered in EBOV infections, with fibrinogen concentration, prothrombin time (PT), and partial thromboplastin time (PTT) significantly increasing over the course of the study (**Supplementary Figure 1** and **Supplementary Table 5**).

Ebola virus-infected domestic ferrets develop pancytopenia and hyperinflammatory responses

We set to investigate ROS accumulation in the context of EVD host responses, with focus on the most salient immune dysregulations known from EVD patients and nonhuman primate (NHP) models[1-3]. We performed flow cytometry and cytokine analyses to characterize cellular and protein host responses in the ferret peripheral blood. Flow cytometry analyses (see **Supplementary Figure 2** for gating strategy) revealed a significant decrease in absolute numbers of $CD8^+$ and $CD4^+$ T lymphocytes, B lymphocytes, and $CD11b^+$ myeloid cells from pre-exposure to dt (**Supplementary Figures 3a-4**), with T $CD4^+$ lymphocytes and $CD11b^+$ myeloid cells also being significantly lower in frequency. The widespread loss of white blood cells was paralleled by a concomitant increase in apoptotic cells over the course of the experiment (**Supplementary Figures 3a-4**). Apoptotic cell numbers significantly increased from pre-exposure to dt for all cell types both in absolute numbers and frequency (with the exception

of the differences in apoptotic CD11b$^+$ cell numbers, which did not reach statistical significance). These results demonstrate that EBOV-infected domestic ferrets develop pancytopenia as previously observed in EVD patients and experimental NHP disease models[1-3]. Descriptive statistics and *p*-values for peripheral blood flow cytometry can be found in **Supplementary Table 6**.

Analyses of serum cytokines revealed an increase in the concentration of all evaluated pro-inflammatory cytokines (interferon alpha [IFNA], C-X-C motif chemokine ligand 8 [CXCL8], and interleukins 6 and 12 [IL6, IL12]) over the course of the experiment (**Supplementary Figures 3b** and **5**). Chemokines related to activation of monocyte/macrophage (C-X-C motif chemokine ligand 10 [CXCL10], and C-C motif chemokine ligands 2 and 4 [CCL2, CCL4]) followed a similar pattern (**Supplementary Figures 3b** and **5**). Among cytokines related to T cell activation, IL17 and tumor necrosis factor (TNF) concentrations were also significantly elevated from pre-exposure (**Supplementary Figures 3b** and **5**). The concentration of IL4, a cytokine characteristic of Th2 responses, remained unaltered similar to during EBOV infection in other animal models[1-3] (**Supplementary Figures 3b** and **5**). Although, to our knowledge, this is the first time that protein responses during EVD have been evaluated in domestic ferrets, the observed changes are in line with the hyperinflammatory response previously reported for EVD patients, in NHPs in an experimental context, and in ferret samples evaluated by RNA expression[1]. The ferrets' hyperinflammatory response and pancytopenia observed in the peripheral blood are consistent with the increased presence of ROS found in the spleens, livers, and kidneys over the course of EBOV infection. Descriptive statistics and *p*-values for serum cytokines can be found in **Supplementary Table 7**.

Supplementary Tables

Supplementary Table 1. Study design: Domestic ferret characteristics at the beginning of the study

Sex	Exposure	Age (mo) at exposure	Weight (kg) at exposure
Male	non-exp	4	1.80
Female	non-exp	4	0.90
Female	non-exp	4	0.86
Female	non-exp	4	0.84
Male	non-exp	4	1.90
Male	non-exp	3	1.42
Male	EBOV	3	1.30
Male	EBOV	3	1.14
Male	EBOV	4	1.62
Male	EBOV	4	1.58
Female	EBOV	4	1.02
Female	EBOV	4	0.84
Female	EBOV	4	0.86
Female	EBOV	4	0.78
Female	EBOV	4	0.90
Female	EBOV	4	0.82

non-exp, non-exposed; EBOV, Ebola virus.

Supplementary Table 2. Study design: Clinical scoring matrix

Parameter	Severity	Score
Appearance	Normal	0
	Pilo-erection, nasal discharge, cyanosis	2
	Visible rash, petechiae or subcutaneous hemorrhage	5
	Hemorrhage from orifices	10
Respiration	Normal (33–36 breaths per min; may vary by animal)	0
	Increased 10% over baseline average	2
	Coughing or sneezing	2
	Labored breathing through mouth	5
Responsiveness	Normal—bright, alert, responsive	0
	Mildly depressed—responds to treats, toys or personnel	2
	Moderately depressed—response requires prodding, loses interest in treats and toys	5
	Severely depressed—no interest in treats and does not respond to staff in the room	10

Total clinical score is calculated as the sum of scores for appearance, respiration and responsiveness.

Animals were euthanized at veterinary discretion or when a total score of ≥10 was reached.

Supplementary Table 3. Study design: Clinical disease assessment, descriptive statistics

	Median (IQR)		
	pre-exp (*n*=16)	3 dpe (*n*=10)	dt (*n*=5)
Weight (kg)	0.960 (0.695)	0.920 (0.555)	0.900 (0.340)
Weight change (%)	None	-1.725 (2.863)	-7.690 (4.805)
Clinical score	0 (0)	0 (0)	12 (7.5)

IQR, interquartile range; pre-exp, pre-exposure; 3 dpe, 3 d post-exposure; dt, day terminal.

Supplementary Table 4. Study design: Virologic assays, descriptive statistics

Assay	Tissue	Median (IQR)			*p*-value		
		pre-exp (*n*=4)	3 dpe (*n*=5)	dt (*n*=5)	pre-exp vs. 3 dpe	pre-exp vs. dt	3 dpe vs. dt
Plaque assay (Log_{10} PFU/mL)	Liver[1]	not analyzed	3.57 (1.705)	8.48 (1.255)	NA	NA	0.0159
	Spleen	not analyzed	3.19 (1)	8.2 (0.58)	NA	NA	0.0079
RT-qPCR (Log_{10} GE/mg)	Liver	0 (0)	2.704 (1.623)	6.822 (0.632)	0.3147	0.0019	0.168
	Spleen	0 (0)	2.167 (1.549)	6.504 (1.152)	0.3147	0.0019	0.168
	Plasma[2]	0 (0)	5.274 (0.613)	12.136 (0.389)	0.0094	0.0001	0.7394

[1]dt (*n*=4); [2]pre-exp (*n*=13), dt (*n*=4). IQR, interquartile range; pre-exp, pre-exposure; 3 dpe, 3 d post-exposure; dt, day terminal; RT-qPCR, real-time reverse transcription polymerase chain reaction; Ge, genome equivalent; NA, not applicable.

Supplementary Table 5. Study design: Serum chemistry and coagulation parameters, descriptive statistics and analysis

Analyte	Median (IQR)			*p*-value		
	pre-exp (*n*=15)	3 dpe (*n*=10)	dt (*n*=5)	pre-exp vs. 3 dpe	pre-exp vs. dt	3 dpe vs. dt
ALB (g/dL)	2.6 (0.3)	2.6 (0.2)	1.9 (0.1)	>0.9999	0.0026	0.0045
ALP (U/L)	88 (41)	73 (23.25)	389 (923)	0.6316	0.0111	0.0007
ALT (U/L)	227 (420)	267.5 (569.5)	2000 (0)	>0.9999	0.0028	0.0143
AMYL (U/L)	23 (7)	23.5 (7)	38 (50)	>0.9999	0.0384	0.0395
AST (U/L)	103 (79)	93 (80.75)	2000 (0)	>0.9999	0.0022	0.0075
BUN (mg/dL)	26 (10)	24.5 (8.25)	77 (46)	>0.9999	0.0063	0.0018
CA (mg/dL)	9.8 (0.4)	9.95 (0.225)	9.4 (2)	0.4697	>0.9999	0.1503
CREA (mg/dL)	0.5 (0.3)	0.5 (0.025)	1 (0.8)	>0.9999	0.0025	0.014
GGT[1] (U/L)	7 (7)	8 (6.5)	68 (77)	>0.9999	0.0017	0.0134
GLU (mg/dL)	136 (30)	176.5 (80.75)	60 (103)	0.0252	0.2663	0.0011
TBIL (mg/dL)	0.3 (0.1)	0.3 (0.025)	3.4 (2.05)	>0.9999	0.0003	0.0061
TP (g/dL)	5.60 (0.8)	5.65 (0.625)	5.60 (0.8)	>0.9999	>0.9999	>0.9999
FBG[2] (g/dL)	155 (76)	201 (104.5)	179.5 (58)	0.0324	0.6585	>0.9999
PTT[3] (s)	17.15 (7.8)	17.8 (5.75)	39.4 (26.9)	>0.9999	0.0074	0.0305
PT[4] (s)	12.1(1.5)	11 (1.25)	46.3 (85.2)	0.2839	0.0171	0.0007

[1] pre-exp (*n*=13), 3 dpe (*n*=9); [2] pre-exp (*n*=13), 3 dpe (*n*=5), dt (*n*=4); [3] pre-exp (*n*=10), 3 dpe (*n*=5); [4] pre-exp (*n*=13), 3 dpe (*n*=5). IQR, interquartile range; pre-exp, pre-exposure; 3 dpe, 3 d post-exposure; dt, day terminal; ALB, albumin; ALP, alkaline phosphatase; ALT, alanine transferase; AMYL, amylase; AST, aspartate aminotransferase; BUN, blood urea nitrogen; CA, calcium; CREA, creatinine; GGT, gamma-glutamyl transferase; GLU, glucose; TBIL, total bilirubin; TP, total protein; FBG, fibrinogen; PTT, partial thromboplastin time; PT, prothrombin time. Mesurements beyond the limit of the dection of the instrument are reported as either the lower or upper detection threshold.

Supplementary Table 6. Ebola virus-infected domestic ferrets develop pancytopenia and hyperinflammatory responses: Blood flow cytometry, descriptive statistics

Population	Cells/mL, median (IQR)						*p*-value		
	pre (n=13)		3 dpe (n=10)		dt (n=4)		pre vs. 3 dpe	pre vs. dt	3 dpe vs. dt
$CD4^+$ T lymphocytes	536523	(1246588)	867465	(1176622)	35457	(98676)	>0.9999	0.0122	0.008
$CD8^+$ T lymphocytes	491929	(661794)	484106	(763579)	118897	(188604)	>0.9999	0.0621	0.0318
B lymphocytes	5485605	(6360207)	4889918	(5657405)	807493	(1081772)	>0.9999	0.0139	0.0169
$CD11b^+$ myeloid cells	752391	(1100283)	1706728	(2324303)	86657	(108766)	0.4136	0.0372	0.0016
Apoptotic population									
$CD4^+$ T lymphocytes	0	(89)	0	(160)	1246	(2741)	>0.9999	0.0069	0.0301
$CD8^+$ T lymphocytes	248	(197)	395	(858)	900	(3674)	>0.9999	0.0242	0.1393
B lymphocytes	1728	(2197)	1390	(6497)	17640	(33319)	>0.9999	0.0419	0.0233
$CD11b^+$ myeloid cells	258	(953)	254	(1932)	3050	(4198)	>0.9999	0.0632	0.0678

Population	Frequency (%), median (IQR)			*p*-value		
	pre (n=13)	3 dpe (n=10)	dt (n=4)	pre vs. 3 dpe	pre vs. dt	3 dpe vs. dt
$CD4^+$ T lymphocytes	7.16 (8.33)	8.00 (5.88)	1.62 (2.30)	>0.9999	0.0429	0.0512
$CD8^+$ T lymphocytes	4.57 (4.83)	5.55 (1.70)	4.95 (2.55)	>0.9999	>0.9999	>0.9999
B lymphocytes	50.31 (16.47)	44.56 (11.75)	46.65 (14.12)	>0.9999	>0.9999	>0.9999
$CD11b^+$ myeloid cells	7.82 (4.39)	13.33 (8.29)	4.03 (1.28)	0.1497	0.0567	0.0007
Apoptotic population						
$CD4^+$ T lymphocytes	0 (0.02)	0 (0.03)	2.61 (13.02)	>0.9999	0.0043	0.008
$CD8^+$ T lymphocytes	0.05 (0.08)	0.06 (0.14)	0.84 (8.53)	>0.9999	0.0098	0.0106
B lymphocytes	0.03 (0.10)	0.03 (0.12)	2.33 (7.83)	>0.9999	0.0122	0.008
$CD11b^+$ myeloid cells	0.04 (0.15)	0.02 (0.09)	3.55 (9.10)	>0.9999	0.015	0.006

IQR, interquartile range; pre-exp, pre-exposure; 3 dpe, 3 d post-exposure; dt, day terminal.

Supplementary Table 7. Ebola virus-infected domestic ferrets develop pancytopenia and hyperinflammatory responses: Serum cytokines and chemokines, descriptive statistics

Cytokine	pg/mL, Median (IQR)			*p*-value		
	pre (n=13)	3 dpe (n=9)	dt (n=5)	pre vs. 3 dpe	pre vs. dt	3 dpe vs. dt
IFNA	61.2 (113.34)	61.58 (104.05)	345.21 (85.83)	>0.9999	0.0081	0.016
IL-12B	123.76 (72.93)	171.63 (104.46)	1140 (0)	0.7838	0.0009	0.033
IL-12	27.43 (49.88)	46.7 (36.49)	191.42 (139.82)	>0.9999	0.0061	0.0205
IL-6	71.2 (0)	71.2 (0)	2860 (2788.8)	>0.9999	0.0034	0.0291
CXCL8	199.67 (443.52)	148.93 (296.335)	2860 (537.04)	>0.9999	0.0079	0.0023
CXCL10	1253.28 (1432.15)	2857 (1499.35)	2857 (0)	0.139	0.0087	0.622
CCL2	322.41 (548.98)	392.67 (630.58)	857 (0)	>0.9999	0.0228	0.1448
CCL4	11.6 (0)	11.6 (0)	121.82 (66.31)	>0.9999	<0.0001	0.0003
IL-2	21.4 (2.31)	21.4 (3.66)	38.11 (24.83)	>0.9999	0.1042	0.4506
TNF	85.2 (22.40)	113.14 (44.64)	248.89 (160.82)	0.5393	0.0013	0.0668
IL-4	83 (0)	83 (0)	83 (9.10)	>0.9999	0.2196	0.0942
IL-17	41.4 (0)	41.4(0)	57.61 (39.16)	>0.9999	0.0114	0.0064

IQR, interquartile range; pre-exp, pre-exposure; 3 dpe, 3 d post-exposure; dt, day terminal. Measurements outside of the range of the established standard curve were set at either the lower or higher detection limit.

Supplementary Table 8. Quantitative immunohistochemistry reveals significant reactive oxygen species tissue accumulation over the course of Ebola virus infection in domestic ferrets: 4-HNE and MPO IHC (% positive area), descriptive statistics

IHC (%)	Organ	Median (IQR)			*p*-value		
		pre-exp (*n*=5)	3 dpe (*n*=5)	dt (*n*=5)	pre-exp vs. 3 dpe	pre-exp vs. dt	3 dpe vs. dt
4-HNE	Spleen	0.0105 (0.3144)	0.0334 (0.0269)	0.4892 (3.4169)	>0.9999	0.0486	0.071
	Liver	0.0132 (0.0664)	0.0445 (0.1444)	0.5689 (2.7613)	>0.9999	0.0175	0.1017
	Kidney	0.0650 (0.0709)	0.0290 (0.4137)	0.2529 (0.7364)	>0.9999	0.4127	0.1687
MPO	Spleen	12.4360 (3.7518)	8.1714 (4.0743)	18.6321 (2.7343)	0.8665	0.1017	0.0044
	Liver	0.1771 (0.1513)	0.1093 (0.1082)	0.4915 (0.4981)	0.6093	0.8665	0.0589
	Kidneys	0.0011 (0.0020)	0.0038 (0.0064)	0.2291 (0.3776)	0.4127	0.0021	0.1687

IQR, interquartile range; pre-exp, pre-exposure; 3 dpe, 3 d post-exposure; dt, day terminal; 4-HNE, 4-hydroxy-2-nonenal; MPO, myeloperoxidase.

Supplementary Table 9. Reactive oxygen species detection by Fe-PyC3A-sensitive magnetic resonance imaging reflects immune dysregulation and oxidative stress: Fe-PyC3A MRI, %CE descriptive statistics and analysis

Organ	Median (IQR)			*p*-value		
	pre-exp (*n*=16)	3 dpe (*n*=10)	dt (*n*=4)	pre-exp vs. 3 dpe	pre-exp vs. dt	3 dpe vs. dt
Spleen	-1.539 (2.753)	0.518 (2.989)	3.624 (7.424)	0.731	0.005	0.090
Bone marrow	0.432 (5.938)	-0.061 (6.218)	-1.798 (3.483)	>0.9999	0.966	>0.9999
Liver	1.908 (4.744)	0.469 (3.082)	4.438 (9.157)	>0.9999	0.499	0.218
Kidneys	-7.346 (9.572)	-6.786 (9.284)	-0.674 (9.211)	>0.9999	0.127	0.204

pre-exposure (pre-exp), 3 d post-exposure (3 dpe), and day terminal (dt). Sample size: *n*=4. IQR, interquartile range.

Supplementary Table 10. Spleens of Ebola virus-infected domestic ferrets are characterized by loss of $CD4^+$ and $CD8^+$ T lymphocytes: Spleen flow cytometry, descriptive statistics

Population	Cells/mL, median (IQR)						*p*-value		
	pre (n=13)		3 dpe (n=10)		dt (n=4)		pre vs. 3 dpe	pre vs. dt	3 dpe vs. dt
$CD4^+$ T lymphocytes	6429986	(6146065)	6810100	(5828745)	2464260	(2084227)	>0.9999	0.04	0.0267
$CD8^+$ T lymphocytes	13749944	(10937363)	31899650	(14286083)	5225524	(9708213)	0.2313	0.5373	0.0056
B lymphocytes	21882770	(18614234)	60450968	(45863646)	20788619	(38008312)	0.1017	>0.9999	0.2691
$CD11b^+$ myeloid cells	23334831	(15177711)	16407033	(9049027)	12562075	(9232544)	>0.9999	0.0589	0.4127
Apoptotic population									
$CD4^+$ T lymphocytes	400	(903)	7425	(23432)	2094	(33357)	0.0173	0.1012	>0.9999
$CD8^+$ T lymphocytes	172872	(140840)	274654	(68385)	54867	(90688)	0.1431	0.5373	0.0027
B lymphocytes	3547	(3140)	20363	(6152)	34163	(47206)	0.0851	0.0056	>0.9999
$CD11b^+$ myeloid cells	24778	(50909)	148033	(133735)	44259	(86089)	0.0216	0.7737	0.3594

Population	Frequency (%), median (IQR)			*p*-value		
	pre (n=13)	3 dpe (n=10)	dt (n=4)	pre vs. 3 dpe	pre vs. dt	3 dpe vs. dt
$CD4^+$ T lymphocytes	3.33 (1.57)	3.11 (1.54)	1.51 (1.59)	>0.9999	0.9666	0.3594
$CD8^+$ T lymphocytes	6.33 (3.10)	12.58 (4.51)	5.69 (3.07)	0.04	>0.9999	0.014
B lymphocytes	9.19 (5.72)	23.56 (7.32)	22.94 (17.41)	0.0267	0.0216	>0.9999
$CD11b^+$ myeloid cells	8.66 (5.66)	8.93 (2.70)	10.91 (7.39)	>0.9999	>0.9999	>0.9999
Apoptotic population						
$CD4^+$ T lymphocytes	0.01 (0.01)	0.15 (0.21)	0.09 (0.9)	0.0472	0.0108	>0.9999
$CD8^+$ T lymphocytes	1.33 (1.02)	1.01 (0.39)	1.10 (0.44)	>0.9999	>0.9999	>0.9999
B lymphocytes	0.01 (0.02)	0.04 (0.03)	0.13 (0.04)	0.4053	0.0019	0.1641
$CD11b^+$ myeloid cells	0.10 (0.22)	0.72 (0.41)	0.36 (0.61)	0.0125	0.1681	>0.9999

IQR, interquartile range; pre-exp, pre-exposure; 3 dpe, 3 d post-exposure; dt, day terminal.

Supplementary References

1 Cross, R. W. *et al.* Comparative transcriptomics in Ebola Makona-infected ferrets, nonhuman primates, and humans. *J Infect Dis* **218 Suppl 5**, S486-S495, doi:10.1093/infdis/jiy455 (2018).

2 Reynard, S. *et al.* Immune parameters and outcomes during Ebola virus disease. *JCI Insight* **4**, e125106 , doi:10.1172/jci.insight.125106 (2019).

3 Wauquier, N., Becquart, P., Padilla, C., Baize, S. & Leroy, E. M. Human fatal Zaire Ebola virus infection is associated with an aberrant innate immunity and with massive lymphocyte apoptosis. *PLoS Negl Trop Dis* **4**, e837 , doi:10.1371/journal.pntd.0000837 (2010).

Supplementary Figure Legends

Supplementary Figure 1: Study design: Serum chemistry and coagulation parameters. Serum chemistries to assess liver (top) and kidney (middle) function and coagulation assays (bottom) (**Supplementary Table 5**). All variables are represented with truncated violin plots, showing the medians (solid line) as a summary measure and interquartile range (IQR, dotted lines). non-exp, non-exposed (beige). 3 dpe, 3 d post-exposure (blue). dt, day terminal (pink). ALT, alanine transferase. AST, aspartate aminotransferase. GGT, gamma-glutamyl transferase. ALP, alkaline phosphatase. BUN, blood urea nitrogen. TP, total protein. PT, prothrombin time. PTT, partial thromboplastin time. * indicates statistical significance ($p < 0.05$).

Supplementary Figure 2. Ebola virus-infected domestic ferrets develop pancytopenia and hyperinflammatory responses: Flow cytometry gating strategy. A traditional gating strategy displays various cellular populations of interest like granulocytes, non-granulocytes, myeloid (CD11b+), and lymphocytes, in whole blood from a ferret with the name and cell frequencies of different cell subsets shown in the plots**.** Total cell populations were calculated based on the counting beads utilized during the sample staining procedure.

Supplementary Figure 3: Ebola virus-infected domestic ferrets develop pancytopenia and hyperinflammatory responses. a) from left to right, graphs of $CD8^{+}$ T lymphocytes, $CD4^{+}$ T lymphocytes, B lymphocytes, and $CD11b^{+}$ myeloid cells over the course of the experiments. **b)** representative graphs and fold change heat map of serum cytokines related to myeloid cell and T/NK cell activation. Circles indicate individual data points. Median and interquartile range (IQR) are also represented. non-exp, not-exposed; 3 dpe, 3 d post-exposure; dt, day terminal.* indicates statistical significance ($p < 0.05$).

Supplementary Figure 4. Ebola virus-infected domestic ferrets develop pancytopenia and hyperinflammatory responses: Line graphs of flow cytometry of the peripheral blood samples. Data from unexposed euthanized animals are shown in beige, data from animals euthanized at 3 dpe are shown in blue, and data from animals euthanized at dt are shown in pink. Circles indicate individual data points. non-exp, not-exposed; 3 dpe, 3 d post-exposure; dt, day terminal.

Supplementary Figure 5. Ebola virus-infected domestic ferrets develop pancytopenia and hyperinflammatory responses: Line graphs of serum cytokine analyses. Data from unexposed euthanized animals are shown in beige, data from animals euthanized at 3 dpe are shown in blue, and data from animals euthanized at dt are shown in pink. Circles indicate individual data points. non-exp, not-exposed; 3 dpe, 3 d post-exposure; dt, day terminal; CCL2, C-C motif chemokine ligand 2; CCL4, C-C motif chemokine ligand 4; CXCL8, C-X-C motif chemokine ligand 8; CXCL10, C-X-C motif chemokine ligand 10; IFNA, interferon alpha; IL2, IL4, IL6, , IL12, IL17, interleukins 2, 4, 6, 12, and 17; NK, natural killer; TNF, tumor necrosis factor; T, T lymphocytes.

Supplementary Figure 6. Reactive oxygen species detection by Fe-PyC3A-sensitive magnetic resonance imaging reflects immune dysregulation and oxidative stress in Ebola virus-infected domestic ferrets. a) line graphs of %CE from Fe-PyC3A-sensitive magnetic resonance imaging (MRI). **b), c), d)** scatter plots of correlation between MRI and quantitative immunohistochemistry (IHC) after 4-hydroxy-2-nonenal (4-HNE) and myeloperoxidase (MPO) staining (Spearman ranks). Data from unexposed euthanized animals are shown in beige, data from Ebola virus-exposed animals euthanized at 3 dpe are shown in blue, and data from exposed

animals euthanized at dt are shown in pink. Circles indicate individual data points. non-exp, not-exposed; 3 dpe, 3 d post-exposure; dt, day terminal.

Supplementary Figure 7. Bone marrow of Ebola-virus-exposed domestic ferrets shows hypocellularity at day terminal. a) hematoxylin and eosin (H&E) histopathology of bone marrow in an unexposed ferret (non-exp) and at 3 d post-exposure (3 dpe) to Ebola virus at day terminal (dt). At pre-exposure and 3 dpe, the bone marrow is histopathologically normal, with no signs of cellular depletion. At dt, the bone marrow presents with hypocellularity in EBOV-infected animals. **b)** graph of semiquantitative depletion score of the bone marrow, showing progressive cellular depletion over the course of the experiment. Data from unexposed euthanized animals are shown in beige, data from Ebola virus-exposed animals euthanized at 3 dpe are shown in blue, and data from exposed animals euthanized at dt are shown in pink. Circles indicate individual data points. * indicates statistical significance ($p < 0.05$).

Supplementary Figure 1

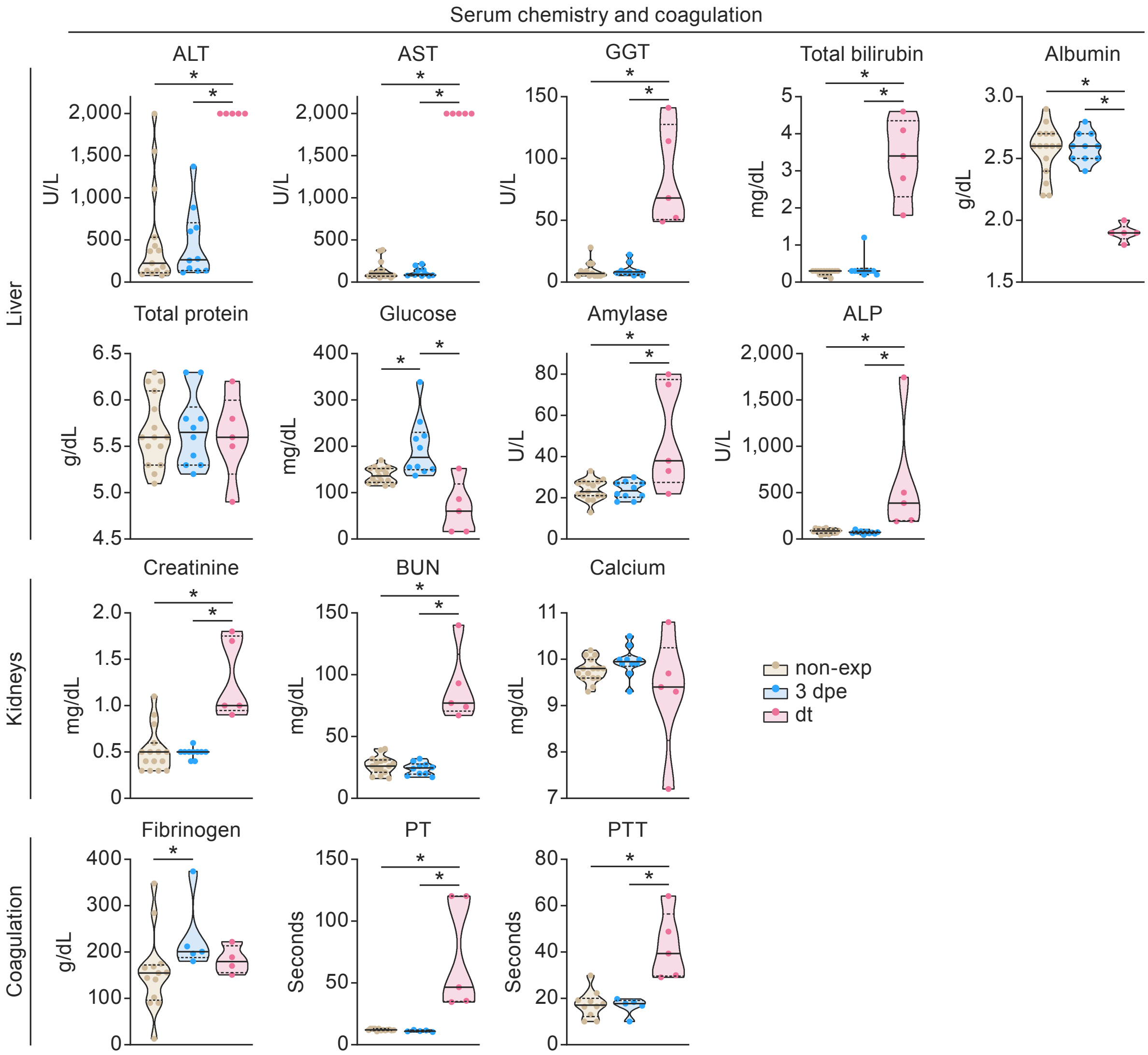

Supplementary Figure 2

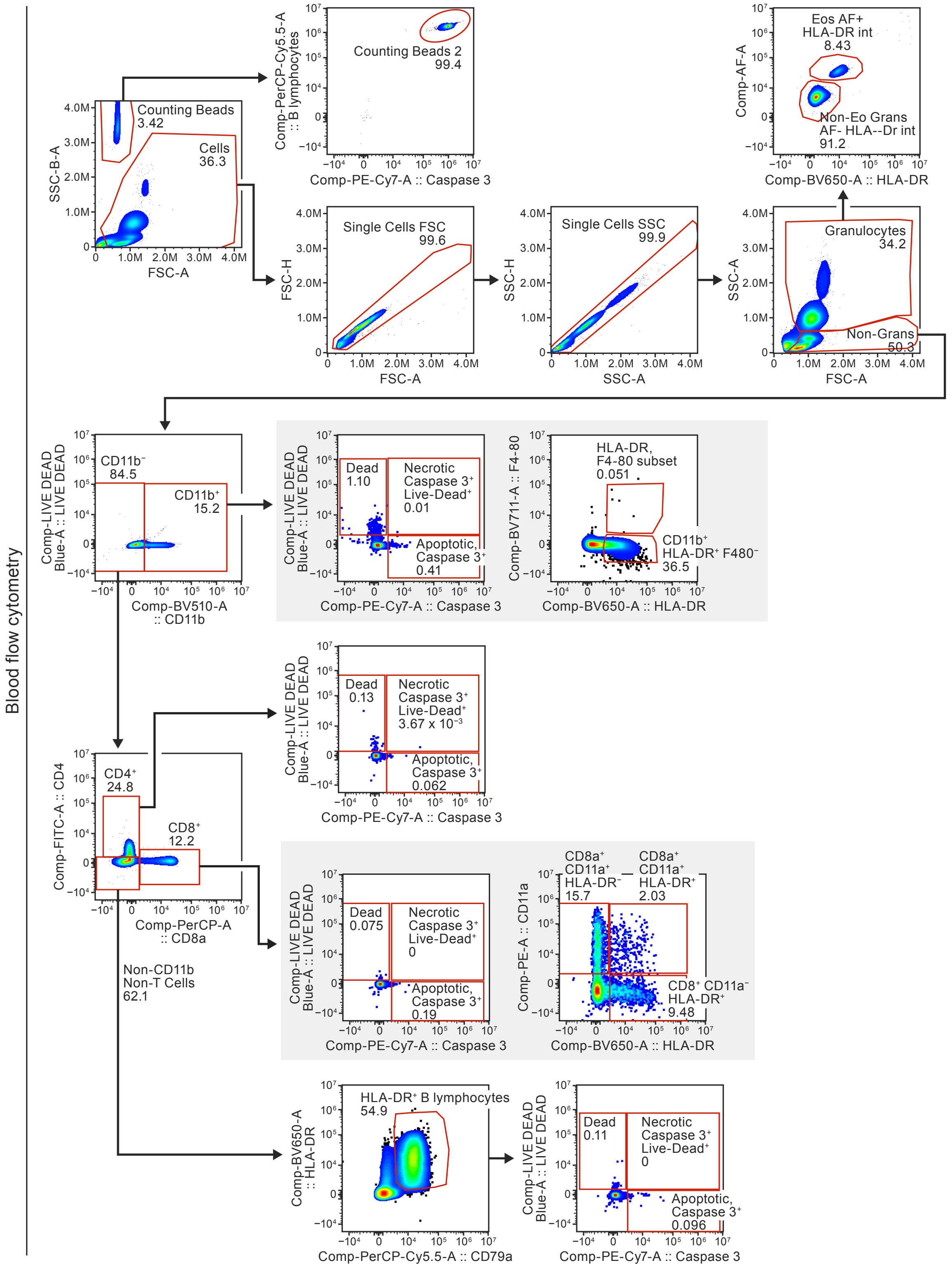

Supplementary Figure 3

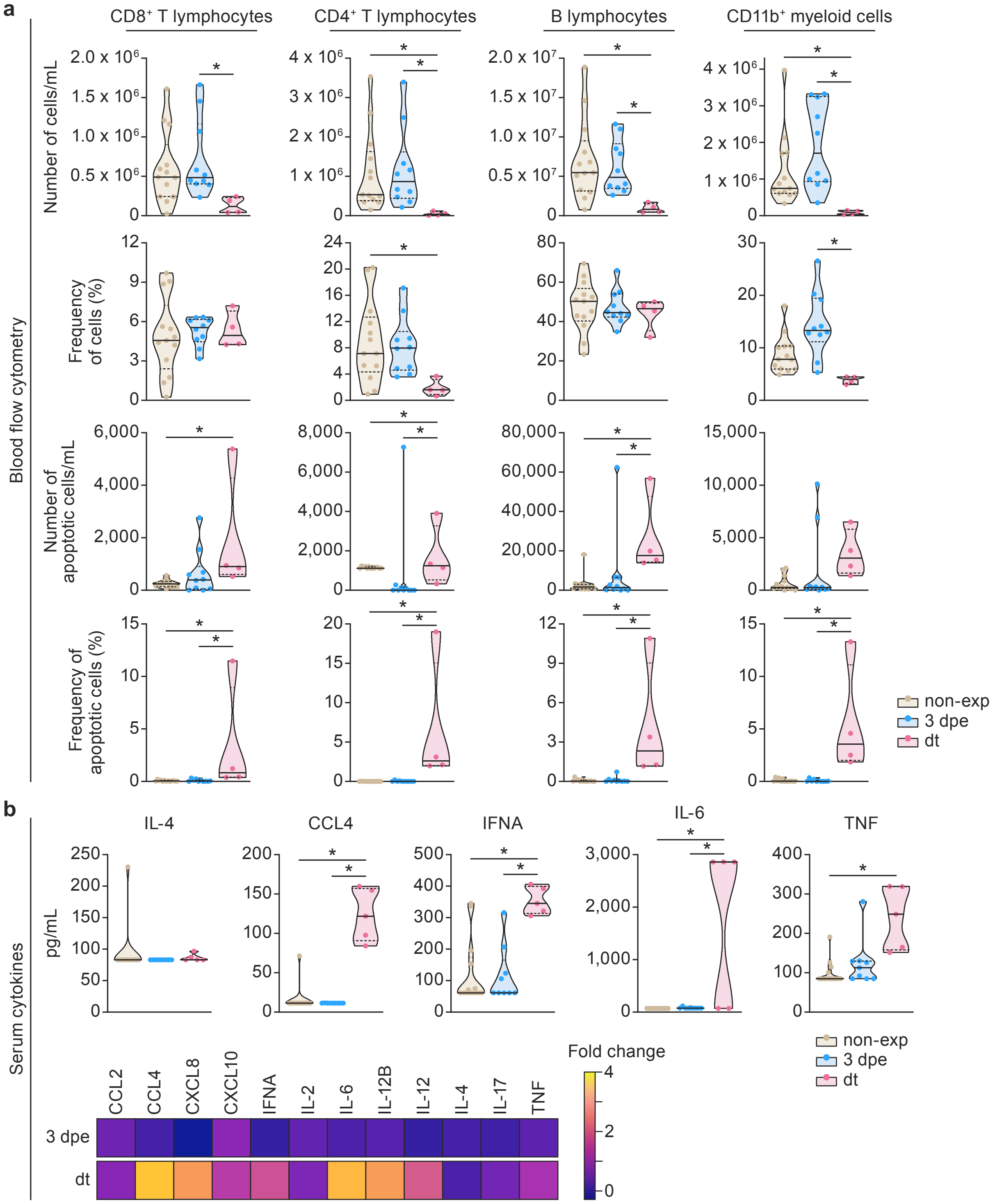

Supplementary Figure 4

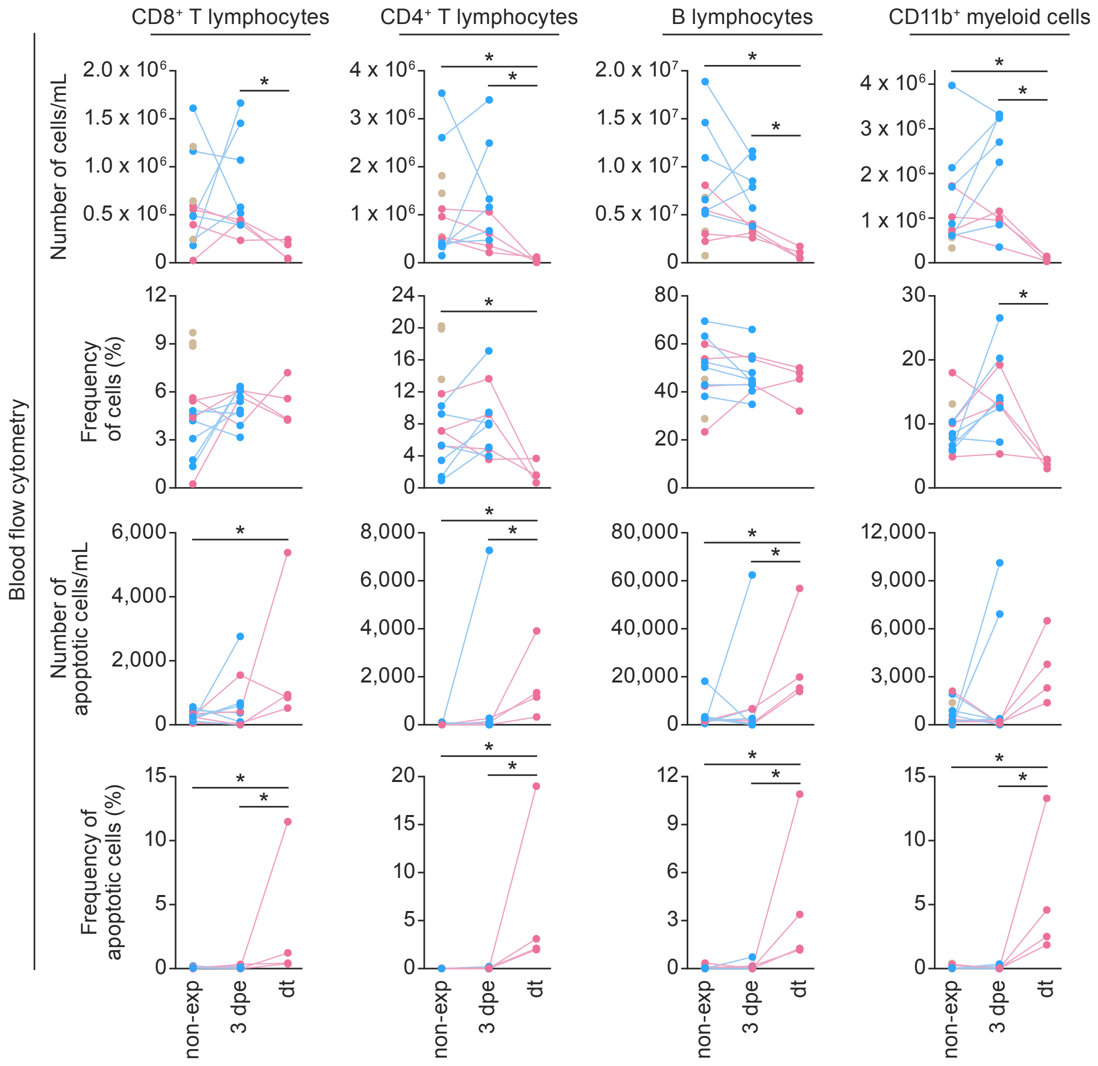

Supplementary Figure 5

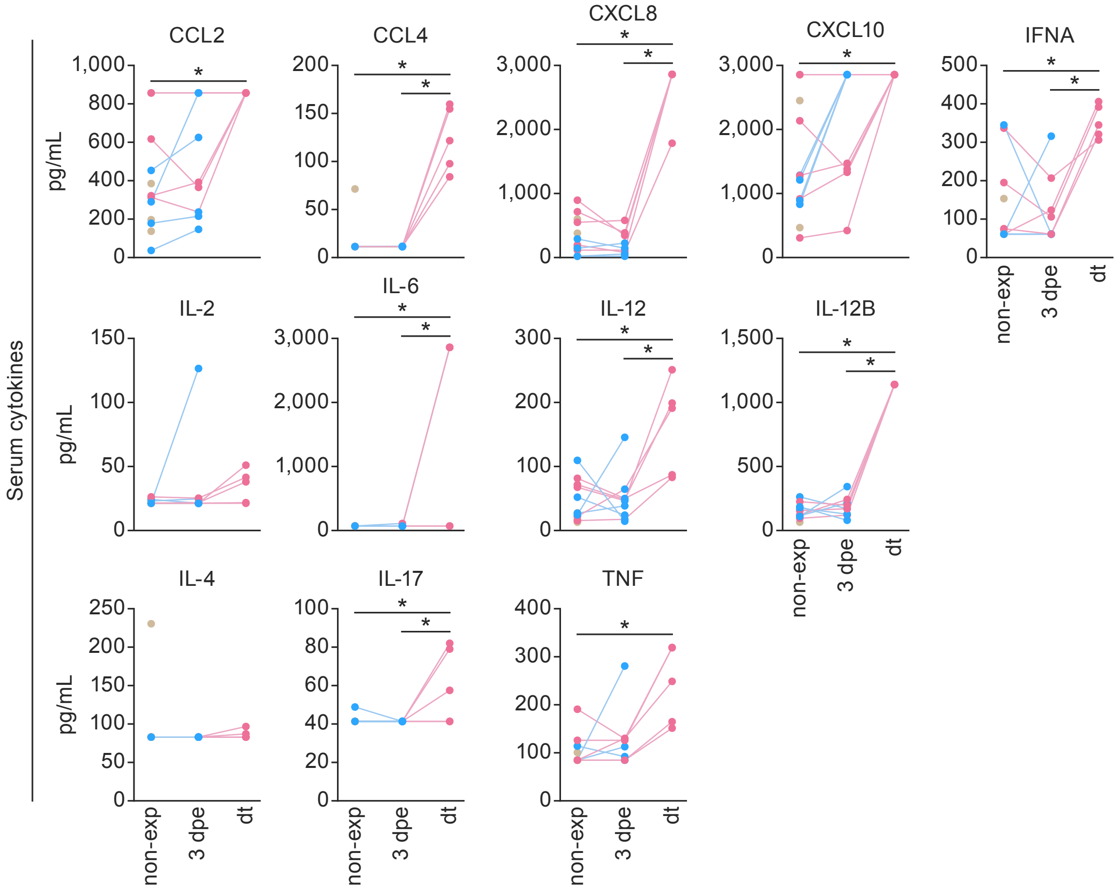

Supplementary Figure 6

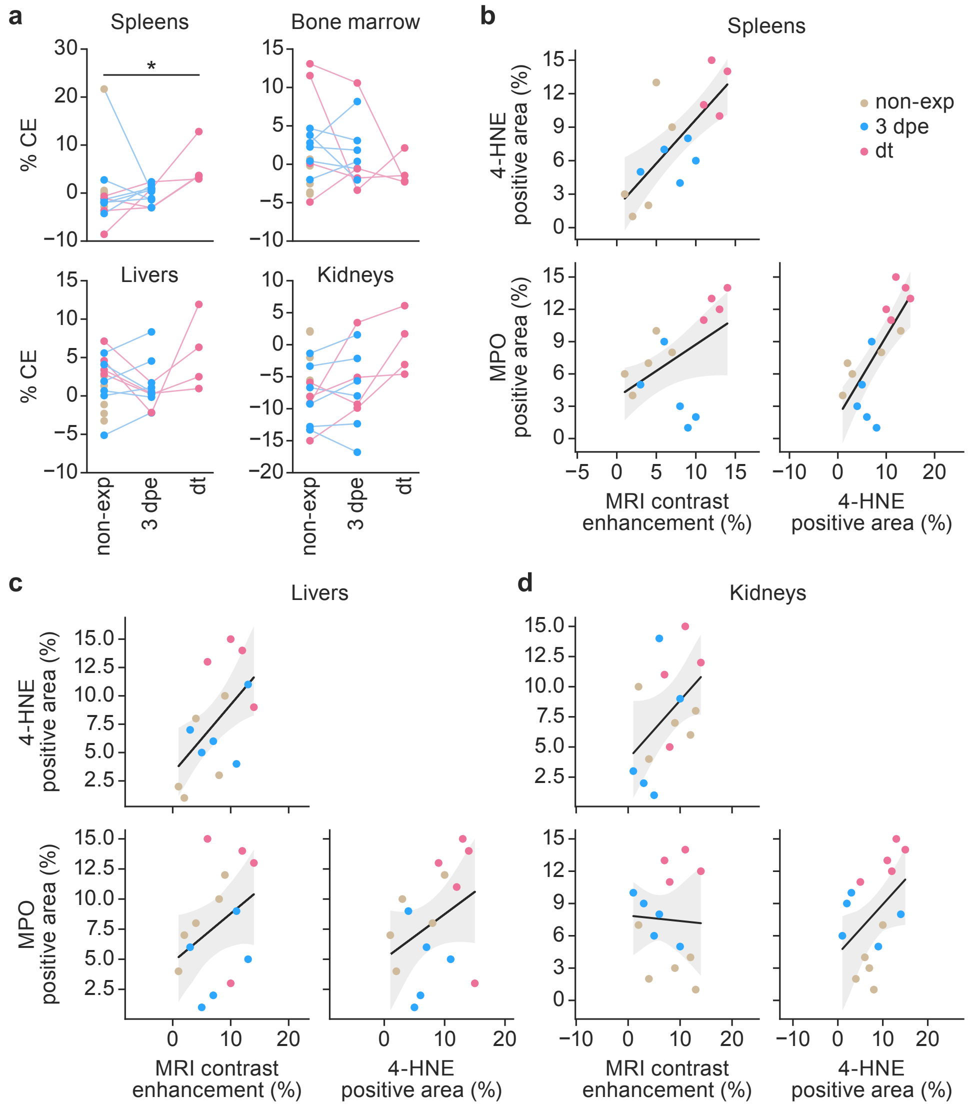

Supplementary Figure 7

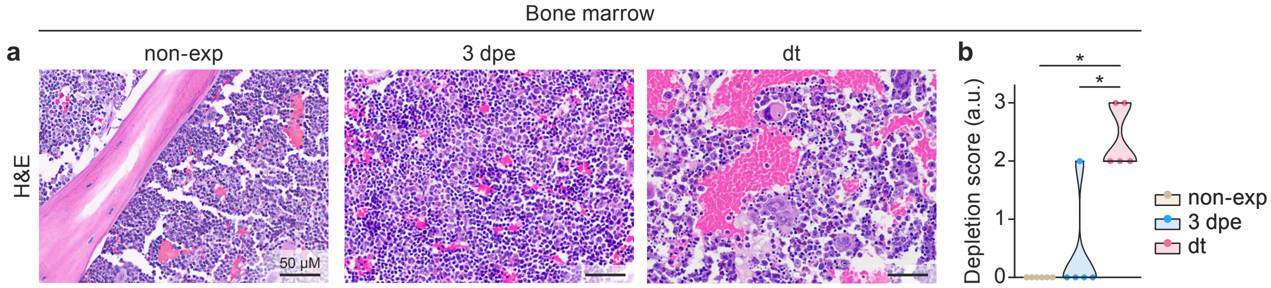

Supplement: Supplementary file 1 — Supplementary figures [file 44303_2025_79_MOESM1_ESM.pdf]
